# *Mycobacterium tuberculosis* WhiB3 Maintains Redox Homeostasis by Regulating Virulence Lipid Anabolism to Modulate Macrophage Response

**DOI:** 10.1371/journal.ppat.1000545

**Published:** 2009-08-14

**Authors:** Amit Singh, David K. Crossman, Deborah Mai, Loni Guidry, Martin I. Voskuil, Matthew B. Renfrow, Adrie J. C. Steyn

**Affiliations:** 1 Department of Microbiology, Centers for Free Radical Biology, AIDS Research, and Emerging Infections and Emergency Preparedness, University of Alabama at Birmingham, Birmingham, Alabama, United States of America; 2 Department of Microbiology, University of Colorado Denver, School of Medicine, Aurora, Colorado, United States of America; 3 Department of Biochemistry and Molecular Genetics, University of Alabama at Birmingham, Birmingham, Alabama, United States of America; Johns Hopkins School of Medicine, United States of America

## Abstract

The metabolic events associated with maintaining redox homeostasis in *Mycobacterium tuberculosis* (*Mtb*) during infection are poorly understood. Here, we discovered a novel redox switching mechanism by which *Mtb* WhiB3 under defined oxidizing and reducing conditions differentially modulates the assimilation of propionate into the complex virulence polyketides polyacyltrehaloses (PAT), sulfolipids (SL-1), phthiocerol dimycocerosates (PDIM), and the storage lipid triacylglycerol (TAG) that is under control of the DosR/S/T dormancy system. We developed an *in vivo* radio-labeling technique and demonstrated for the first time the lipid profile changes of *Mtb* residing in macrophages, and identified WhiB3 as a physiological regulator of virulence lipid anabolism. Importantly, *MtbΔwhiB3* shows enhanced growth on medium containing toxic levels of propionate, thereby implicating WhiB3 in detoxifying excess propionate. Strikingly, the accumulation of reducing equivalents in *MtbΔwhiB3* isolated from macrophages suggests that WhiB3 maintains intracellular redox homeostasis upon infection, and that intrabacterial lipid anabolism functions as a reductant sink. *MtbΔwhiB3* infected macrophages produce higher levels of pro- and anti-inflammatory cytokines, indicating that WhiB3-mediated regulation of lipids is required for controlling the innate immune response. Lastly, WhiB3 binds to *pks2* and *pks3* promoter DNA independent of the presence or redox state of its [4Fe-4S] cluster. Interestingly, reduction of the apo-WhiB3 Cys thiols abolished DNA binding, whereas oxidation stimulated DNA binding. These results confirmed that WhiB3 DNA binding is reversibly regulated by a thiol-disulfide redox switch. These results introduce a new paradigmatic mechanism that describes how WhiB3 facilitates metabolic switching to fatty acids by regulating *Mtb* lipid anabolism in response to oxido-reductive stress associated with infection, for maintaining redox balance. The link between the WhiB3 virulence pathway and DosR/S/T signaling pathway conceptually advances our understanding of the metabolic adaptation and redox-based signaling events exploited by *Mtb* to maintain long-term persistence.

## Author Summary

Currently, approximately one-third of the world's population is latently infected with *Mycobacterium tuberculosis* (*Mtb*), the bacterium that causes tuberculosis (TB). A central question in TB research is to identify the mechanisms that allow the organism to persist for long periods of time in humans. The mycobacterial cell wall has a high lipid content and contains several important lipid groups, including poly- and di-acyltrehaloses (PAT/DAT), sulfolipids (SL-1), and phthiocerol dimycocerosates (PDIM). These lipids are produced and actively secreted during infection to subvert the host immune system, eventually leading to *Mtb* persistence. We have discovered that the regulatory protein WhiB3 controls the differential production of PAT, DAT, SL-1, and PDIM and the storage lipid triacylglycerol (TAG) in response to fluctuations in the intracellular redox environment. We demonstrated that WhiB3 directly regulates lipid production by binding to the promoter regions of lipid biosynthetic genes in a redox-dependent manner. We also discovered that through this regulatory process, WhiB3 controls fatty acid metabolism and maintains intracellular redox homeostasis by channeling toxic reducing equivalents into lipid anabolism. Thus, our results suggest that *Mtb* lipid anabolism functions as a reductant sink to neutralize the reductive stress associated with the catabolism of host lipids during infection. These findings may serve as a model foundation for how pathogens adjust their metabolism to cope with stresses encountered during infection.

## Introduction

The success of *Mtb* as a remarkably effective pathogen is due to the ability of the bacilli to latently infect ∼2 billion people world wide [1]. The metabolic events necessary for *Mtb* to enter, maintain and emerge from a latent infection are poorly understood, but are crucial towards the development of new drugs and vaccines, primarily because latent *Mtb* is in a state of drug unresponsiveness. Persistent infection is a complex interplay between the host immune system and bacterial virulence mechanisms, and little is known about the environmental signals and regulatory cascades involved in the regulation of specific bacterial component involved in this process.

The role of *Mtb* cell wall polyketide lipids has received wide attention because it has been demonstrated that surface exposed polyketides such as PDIM and PGL-tb interact with the host to regulate *Mtb* virulence [2],[3]. An earlier study hypothesized that the failure of *Mtb* to induce a complex protective response against oxidative stress is because its complex cell wall lipids act as a constitutive defense mechanism to withstand oxidative insult [4]. Indeed, cell wall components such as phenolic glycolipid (PGL-1) [2], mycolic acids and PDIM were shown to play a role in protecting *Mtb* against redox stress and antibiotics [5]–[7] whereas other lipids (*e.g.*, SL-1) exert immunomodulatory effects [8]. Complex lipids are also thought to regulate the degree of virulence, for example hypervirulence of the W-Beijing strains was linked to the production PGLs [3].

Recent studies have demonstrated that TAG accumulates in *Mtb* cells during hypoxia, nitric oxide (NO) exposure [9], in the sputum of TB patients [10], and in *Mtb* Beijing strains [11]. Subsequently, it was proposed that *Mtb* TAG functions as a preferred carbon source during long-term persistence and reactivation [9]. However, it has also been suggested that bacterial TAG may function as a sink for reducing equivalents [12]. Collectively, these studies point toward a complex mechanism involving *Mtb* lipids to effectively adapt and respond to host generated redox stress. Identity of an *Mtb* regulator that integrates environmental redox stress signals with the production of bioactive lipids to modulate pathogenesis is not known, and will be an important contribution to the TB field.

Previously, we have shown that *Mtb* WhiB3 controls virulence in two animal models of TB [13]. WhiB orthologues have been implicated in a variety of pathways including sporulation, pathogenesis, cell division [14], oxidative stress [15], and antibiotic resistance [16]. *Mtb whiB3* expression was shown to be induced in phagosomes [17] and during infection of mouse lungs [18]. The pathology defect exhibited by *MtbΔwhiB3* in the mouse model [13] as well as the altered colony rugosity and growth properties of *MtbΔwhiB3* on acetate [19] suggest that WhiB3 is involved in maintaining redox homeostasis by regulating fatty acid metabolism in *Mtb*.

A fundamentally important question remains yet unanswered: What is the mechanism by which WhiB3 contributes to *Mtb* persistence and virulence? In this study we exploited transmission (TEM) and scanning electron microscopy (SEM) and studied the ultrastructure of wild type (wt) *Mtb* and *MtbΔwhiB3*. We comprehensively analyzed the lipid content of wt *Mtb* and *MtbΔwhiB3* under oxido-reductive stress conditions, and upon infection of macrophages. Importantly, we examined the intracellular redox state of wt *Mtb* and *MtbΔwhiB3* cells derived from infected macrophages and analyzed the outcome of *whiB3* loss on the host immune response. Finally, we examined the ability of WhiB3 to bind the promoter regions of polyketide biosynthetic genes in a redox-dependent manner. Our results provide mechanistic insight into the metabolic events required for maintaining redox homeostasis in *Mtb* during infection.

## Results

### *Mtb* WhiB3 is essential for maintaining cell shape and size

**Figure 1:**
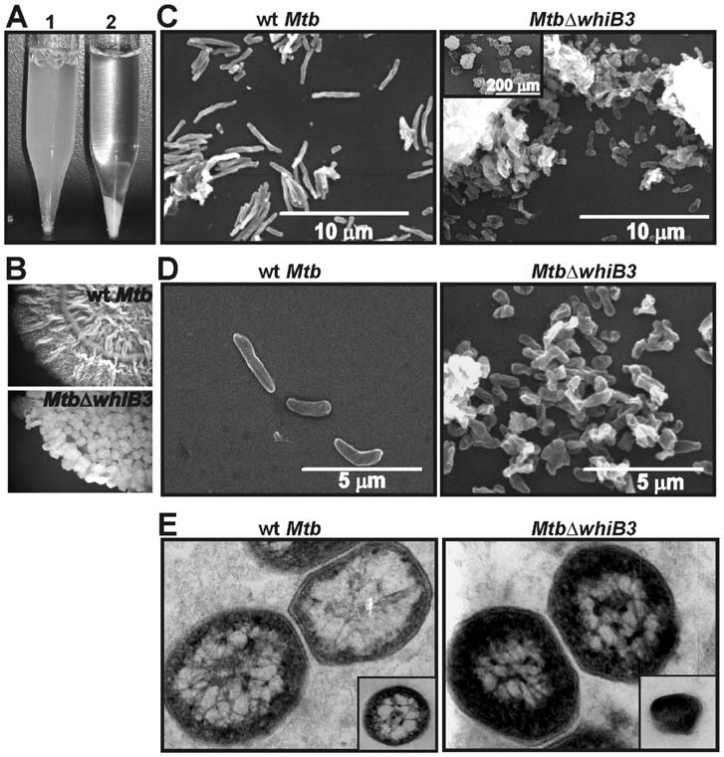
*Mtb* WhiB3 regulates cell shape and size. (A) wt *Mtb* and *MtbΔwhiB3* were cultured in 7H9 liquid media containing 0.1% Tween 80 till stationary phase. At an OD_600 nm_ = 4.5 the aggregation phenotype was examined by allowing the cells to settle for 10 min. *MtbΔwhiB3* cells settled rapidly whereas wt *Mtb* remained dispersed (B) Spot colony rugosity of wt *Mtb* and *MtbΔwhiB3* was analyzed by spotting identical volumes (30 µl) and equal number of cells (3×10^6^) on Dubos complete medium. Cells were allowed to grow for 4 weeks and photographs were taken at 7× magnification using a Zeiss stereo microscope. (C and D) SEM analysis demonstrating that WhiB3 affects cell length. Low magnification SEM (C, *inset*) illustrates the severe clumping of *MtbΔwhiB3* cells. Approximately 5 SEM fields (10 cells/field) were analyzed to determine the cell sizes of wt *Mtb* and *MtbΔwhiB3*. (E) TEM analysis showing hyperstaining of *MtbΔwhiB3* cells as compared to wt *Mtb* cells. *Inset*; low magnification image.

*MtbΔwhiB3* cells cultured in liquid media containing tween-80 displayed significant clumping and aggregation specifically in the late stationary growth phase *i.e* OD_600 nm_ = 3.0 (Fig. 1A, tube 2). To investigate the possibility that the observed aggregation was not due to the efficient utilization of Tween-80 by the *MtbΔwhiB3* mutant, we examined the aggregation phenotype in liquid media containing tyloxapol (a non-hydrolyzable dispersing agent). Similar aggregation of *MtbΔwhiB3* was observed when cultured in the liquid media containing tyloxapol (data not shown). On solid media, *MtbΔwhiB3* generated a disordered colony organization, suggesting the loss of cording (Fig. 1B). On the SEM grid, *MtbΔwhiB3* cells were organized in discrete clusters such that it was difficult to identify individual cells for morphological analysis (Fig. 1C *inset* and 1D). Wt *Mtb* appeared as long rods (average length, ∼2.8±0.8 µm) whereas *MtbΔwhiB3* cells were significantly reduced in length (average length, ∼0.9±0.05 µm) (Fig. 1C and 1D), and appeared irregular and shrunken. In our TEM analysis, we consistently observed poor contrast and hyper-staining of *MtbΔwhiB3* cells compared to wt *Mtb* cells (Fig. 1E), reflecting clear perturbations of the cell wall composition. These results confirmed the essential role of WhiB3 in maintaining the appropriate cell size, shape, and surface architecture of *Mtb*.

### *Mtb* WhiB3 modulates the biosynthesis of complex virulence lipids

**Figure 2:**
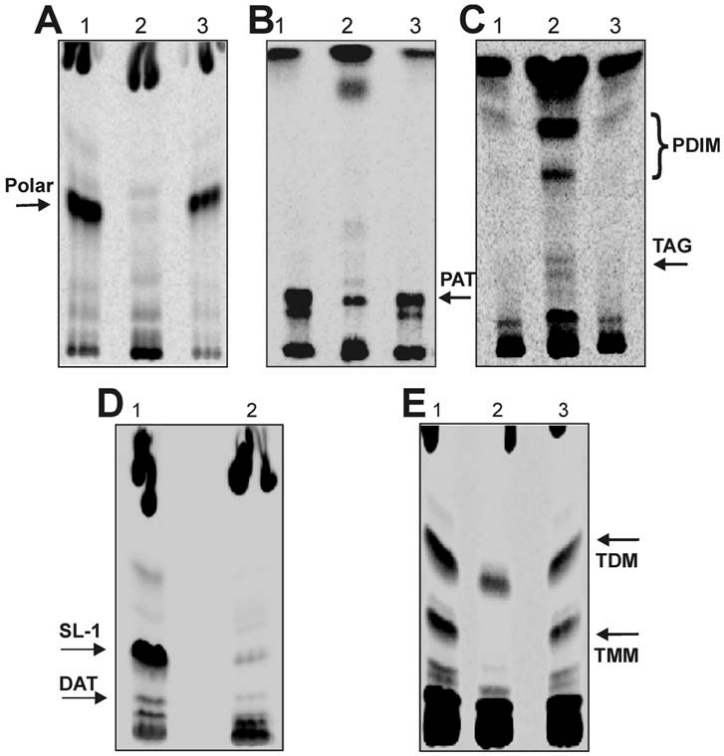
*Mtb* WhiB3 regulates biosynthesis of complex virulence lipids. Wt *Mtb*, *MtbΔwhiB3*, and *MtbΔwhiB3tetRO:whiB3* (*Comp*.) were cultured in 7H9 liquid media containing 0.1% Tween 80, and total lipids labeled using [^14^C] propionate. In each lane, silica TLC plates were loaded with 100,000 cpm of [^14^C] propionate-derived total lipids and various lipid fractions were analysed. (A) Polar lipid fraction was resolved using 10% methanol in chloroform as the solvent. (B) PAT lipids were analyzed by developing TLC plates in petroleum ether∶acetone (92∶8). (C) PDIM and TAG were analyzed by developing TLC plates in petroleum ether∶acetone (98∶2). (D) SL-1 and DAT were analyzed by developing TLC plates in chloroform∶ethanol∶water (90∶10∶1). (E) TMM and TDM were analyzed by loading 100,000 cpm of [1, 2-^14^C] acetate-derived total lipids on a silica TLC plate and developed using chloroform∶methanol∶acetone∶acetic acid (80∶20∶6∶1). Lanes (1) wt *Mtb*, (2) *MtbΔwhiB3* and (3) complemented *MtbΔwhiB3tetRO:whiB3* strain (*Comp*). Phosphorimaging (imageQuant software; GE Healthcare) were used to examine the relative distribution of ^14^C among the lipid fractions.

In order to confirm that the altered *in vitro* growth morphology phenotype of *MtbΔwhiB3* was due to defective cell envelope lipid composition, we biochemically analyzed the lipid content of wt *Mtb* and *MtbΔwhiB3* cells. We performed our lipid analysis using well dispersed cultures of *MtbΔwhiB3* grown to OD_600 nm_ = 1.5. Lipids containing methyl-branched fatty acids of wt *Mtb* and *MtbΔwhiB3* cells were metabolically labeled using ^14^C propionate as a radiotracer. Interestingly, wt *Mtb* incorporates 20–25% of the administered ^14^C into total lipid whereas *MtbΔwhiB3* incorporates only 10–15% in 24 h. Since wt *Mtb* and *MtbΔwhiB3* showed similar growth characteristics as judged by the increase in cell mass and CFU analysis [data not shown], we normalized for the difference in lipid biosynthetic ability of *MtbΔwhiB3* by loading equal radioactive counts per minute (cpm) of ^14^C incorporated total lipids and analyzed the samples via thin layer chromatography (TLC). A detailed analysis of the ^14^C distribution pattern showed significant alterations among PAT, DAT, SL-1, PDIM and TAG in *MtbΔwhiB3* (Table 1). As shown in Fig. 2A, we observed that the polar lipid fraction was absent in *MtbΔwhiB3*. Consistent with this observation, *MtbΔwhiB3* showed a defect in the production of methyl-branched polar lipids PAT, DAT and SL-1 (Fig. 2B, D). Intriguingly, we observed a 5-fold increase in the labeling of PDIM, and minor accumulation of TAG in *MtbΔwhiB3* compared to wt *Mtb* (Fig. 2C). Similar changes were observed for PAT and PDIM isolated from early logarithmic phase cultures (OD_600 nm_ = 0.6) (Fig. S1). The defective production of surface exposed polar lipids such as SL-1, PAT and DAT along with the accumulation of non-polar lipids such as PDIM, suggests that the aggregation phenotype exhibited by *MtbΔwhiB3* cells is due to increased hydrophobicity of the cell surface.

**Table 1:** Relative distribution of ^14^C (%) among the lipid classes derived from ^14^C propionate incorporation in wt *Mtb* and *MtbΔwhiB3*.

Lipid class	Wt *Mtb* (%)	*MtbΔwhiB3* (%)
DIM A	10	50
DIM B	5	20
SL-1	45	4
PAT	29	10
DAT	5	1
TAG	1	5
Highly polar^a^	5	10

To calculate the relative distribution of ^14^C, phosphorimaging measurements were performed on bands corresponding to the different lipids. Results presented are representative of three independent experiments.

a These lipids remained at the origin of the TLC; the solvent system was 10% methanol in chloroform.

Next, we examined the mycolic acid profiles of wt *Mtb* and *MtbΔwhiB3* using [1],[2] ^14^C acetate as tracer. Both wt *Mtb* and *MtbΔwhiB3* incorporated 25–30% of ^14^C into total lipids in 24 h. No quantitative or qualitative differences between wt *Mtb* and *MtbΔwhiB3* were observed in the case of arabinogalactan linked mycolates (data not shown). However, we observed a 2-fold decrease in the labeling of α,α′-trehalose di-mycolate (TDM) and 5-fold decrease of α,α′-trehalose mono-mycolate (TMM) in *MtbΔwhiB3* cells (Fig. 2E). Importantly, complementation using wt *Mtb whiB3* restored the cell wall lipid defect of *MtbΔwhiB3*. We also detected an altered lipid profile from the culture filtrate similar to the cell pellet of *MtbΔwhiB3*, ruling out the contribution of a defect in the secretion of lipids in the observed phenotype (data not shown).

In sum, our lipid analysis demonstrated that *MtbΔwhiB3* is defective in the production of surface associated virulence lipids. An important discovery is that *MtbΔwhiB3* accumulates PDIM (and TAG), a finding that has not been reported for any *Mtb* mutant to date. These data strongly suggest that the prior pathology defect exhibited by the *Mtb*Δ*whiB*3 strain in mice [13] was in part due to an altered repertoire of bioactive polyketides.

### *Mtb* WhiB3 modulates the biosynthesis of complex virulence lipids in a redox-dependent manner

To date, the identity of environmental stimuli regulating the production of complex *Mtb* virulence polyketides or lipids remains unknown. We have previously proposed that *Mtb* WhiB3 acts as an intracellular redox sensor involved in maintaining redox balance by regulating central metabolism [19]. To confirm that WhiB3 couples the intracellular redox environment of *Mtb* with lipid synthesis, wt *Mtb* and *MtbΔwhiB3* lipid profiles were analyzed under defined redox stress conditions.

**Figure 3:**
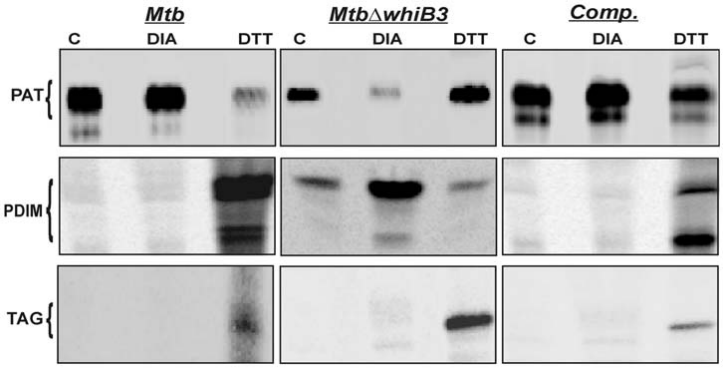
*Mtb* WhiB3 regulates biosynthesis of pathogenic lipids in response to oxido-reductive stress. Complex virulence lipids were analyzed by metabolic labeling using [^14^C] propionate under oxidizing (5 mM diamide) or reducing (5 mM DTT) conditions for 24 h. PAT, PDIM and TAG production was analyzed by spotting equal count in each case (50,000 cpm) on silica TLC and resolved using solvent systems as described in Figure 2. C; untreated cells.

We chose to compare the effect of an altered *Mtb* intracellular redox environment on the assimilation of propionate into PAT and PDIM production. Wt *Mtb* and *MtbΔwhiB3* cells from the logarithmic growth phase were exposed to 5 mM diamide or DTT, followed by radiolabeling with ^14^C propionate. Interestingly, in the DTT containing medium, wt *Mtb* incorporated 3-fold less ^14^C into total lipid as compared to cells grown in control (7H9) or diamide containing medium. However, in the case of *MtbΔwhiB3*, we observed a 1.5 and 3-fold reduction in the incorporation of ^14^C during growth in DTT or diamide containing medium, respectively, as compared to the control. Since, 5 mM DTT or diamide has no influence on the growth of wt *Mtb* or *MtbΔwhiB3* (as judged by spot colony phenotype; data not shown), we normalized the difference in rate of ^14^C incorporation by loading equal cpm on the TLC plates. First, we analyzed wt *Mtb*. Fig. 3 demonstrates that diamide exposed wt *Mtb* cells produces 5-fold more PAT as compared to DTT exposed *Mtb* cells. In contrast, DTT exposed *Mtb* cells incorporate 10-fold more propionate into PDIM as compared to diamide treated cells (Fig. 3). Next, we analyzed how reduced or oxidized *MtbΔwhiB3* cells affect propionate assimilation into PAT and PDIM. We demonstrated that in *MtbΔwhiB3*, the production of PAT and PDIM in response to diamide and DTT was exactly the opposite of wt *Mtb* (Fig. 3).

Since production of TAG is slightly enhanced in *MtbΔwhiB3*, we analyzed the assimilation of ^14^C propionate into TAG under oxidizing and reducing conditions. Strikingly, Fig. 3 shows a 3-fold induction of TAG production in DTT-exposed *MtbΔwhiB3* as compared to wt *Mtb*. Consistent with these results, we observed similar accumulation of TAG in DTT-exposed *MtbΔwhiB3* when labeled using [1],[2] ^14^C acetate as a tracer (Fig. S2). Importantly, the altered lipid profile of *MtbΔwhiB3* upon modulation of the intracellular redox environment was restored to wt *Mtb* lipid levels by complementing *MtbΔwhiB3* cells with wt *whiB3*. The implications of these findings are significant and suggest that intracellular oxidative or reductive stress in *Mtb* modulates anabolism of diverse polyketides required for virulence, as well as TAG, which might be essential for long-term persistence and reactivation.

### *Mtb* WhiB3 directs the synthesis of virulence lipids within macrophages

**Figure 4:**
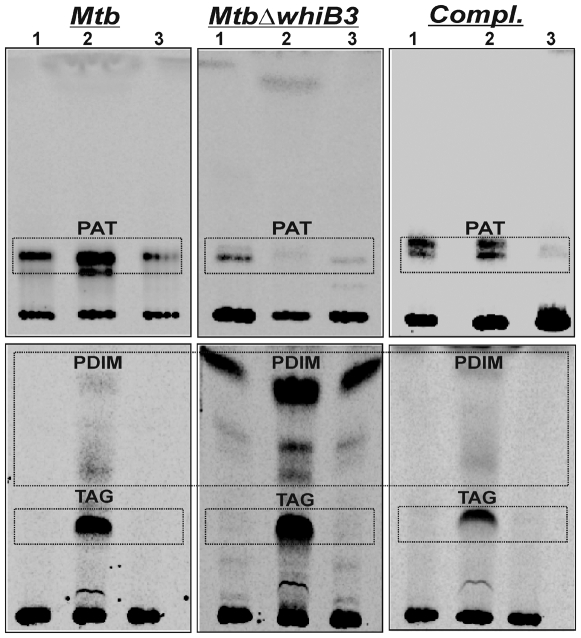
*Mtb* WhiB3 regulates biosynthesis of pathogenic lipids within resting macrophages. Raw 264.7 cells (5×10^8^) were independently infected with lightly sonicated wt *Mtb*, *MtbΔwhiB*3 and *MtbΔwhiB3 tetRO:whiB3* at a MOI of 10∶1 for 4 h. Total lipids from *Mtb* growing within macrophages were metabolically labeled by the addition of [^14^C] propionate for 2 days, and analyzed by TLC (see Materials and Methods). PAT, PDIM and TAG were analyzed by loading equal counts (20,000 cpm) on TLC plates, and developed using solvents as described in Fig. 2 legend. Lipids isolated from *Mtb* growing in Lane (1) 7H9 medium, (2) Raw264.7 and (3) DMEM medium.

Much of the existing *Mtb* literature is derived from the transcriptional response of lipid biosynthetic genes *in vivo*, and does not reflect the exact physiological level and composition of virulence lipids produced during infection. To gain insight into *Mtb* metabolic and redox-mediated events during infection, we performed the first assessment of lipid profile changes of *Mtb* residing in Raw 264.7 macrophages. We infected macrophages using well dispersed, logarithmically grown cultures of *MtbΔwhiB3*. Radiolabeling of lipids was performed using ^14^C propionate as a tracer. Wt *Mtb* and *MtbΔwhiB3* showed 4-fold reduction in the incorporation of ^14^C into total lipids within macrophages as compared to cells cultured *in vitro* (7H9 or DMEM). With the newly developed [^14^C]-propionate labeling and extraction method, we demonstrated that wt *Mtb* within macrophages assimilates 2, 3 and 10- fold more propionate into SL-1, PAT and TAG, respectively, as compared to cells cultured *in vitro* (7H9) (Fig. 4 and Fig. S3). Strikingly, *MtbΔwhiB3* cells cultured *in vivo* incorporated 2-fold less ^14^C propionate into PAT and SL-1 (Fig. 4 and Fig. S3) and a 3 and 15-fold increased incorporation into PDIM and TAG, as compared to cells cultured *in vitro* (Fig. 4). Lipids derived from macrophages were successfully removed by washing infected cells twice in methanol (see Materials and Methods). As a control, uninfected macrophages were metabolically labeled with [^14^C]-propionate and treated in a similar manner as infected macrophages to ensure that lipids were derived from intracellular bacteria and not macrophages (see Materials and Methods).

Further, using Fourier Transform Ion Cyclotron Resonance Mass Spectrometry (FT-ICR MS) we analyzed the total lipid profile of *Mtb* as previously described [20]. As anticipated, FT-ICR MS analysis of total lipid extracts derived from *Mtb* and *MtbΔwhiB3* after infection of macrophages resulted in the identification of complex lipid species including phosphatidylinositol mannosides (PIMs), PDIM and SL-1 (Fig. S4). Differences in phosphatidyl inositol (PI) and phosphatidyl inositol mannoside (PIM) production by wt *Mtb* and *MtbΔwhiB3* within macrophage cells were not observed (Fig. S4A). However, the SL-1 profile was noticeably altered in lipid samples from *MtbΔwhiB3* infected macrophages (Fig. S4B). In fact, the SL-1 spectrum, which is comprised of various lipoforms differing by 14 mass units between *m/z* 2400–2600 [20], was completely absent in the *MtbΔwhiB3* sample (Fig. S4B). Furthermore, consistent with our metabolic labeling data, we did observe PDIM in crude lipid extracts derived from *in vivo* grown wt *Mtb* and *MtbΔwhiB3* (Fig. S4C). FT-ICR MS analysis of lipids isolated from wt *Mtb* and *MtbΔwhiB3* cultured *in vitro* also demonstrated a similar profile to the *in vivo* data (not shown). Importantly, as shown by metabolic labeling (Fig. 4) and FT-ICR MS analysis (Fig. S4), complementation of *MtbΔwhiB3* with *whiB3* restored the wt lipid profile inside macrophages.

In sum, data generated using two independent techniques provide novel insight into the metabolic adaptation of *Mtb* during growth *in vitro* and *in vivo*. Lastly, WhiB3 was identified as an important physiological regulator of PAT, DAT, SL-1, PDIM and TAG anabolism *in Mtb*.

### WhiB3 modulates propionate toxicity in *Mtb*

**Figure 5:**
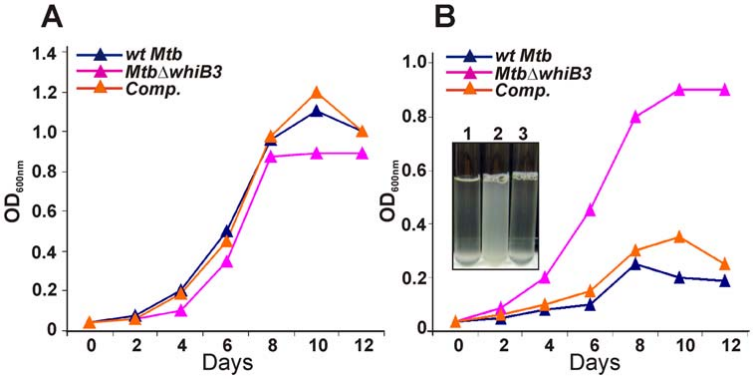
*Mtb* WhiB3 modulates propionate toxicity. Cultures of *Mtb* were synchronized to OD_600 nm_ = 0.04 and grown in 7H9 broth containing 10 mM (A) or 20 mM (B) sodium propionate. Culture turbidity (OD_600 nm_) was measured at the indicated time points. Results are representative of two independent experiments showing similar results. *Inset*; liquid cultures of *wt Mtb* (1), *MtbΔwhiB*3 (2) and *Comp* (3) grown for 10 days in 20 mM propionate.

Since it was proposed that methyl-branched polyketide production functions as a mechanism to remove toxic levels of propionate *in vivo* [21], we analyzed the growth of *MtbΔwhiB3* on propionate containing medium. Wt *Mtb* and *MtbΔwhiB3* grew virtually identically in media containing 10 mM of propionate as a sole carbon source (Fig. 5A). However, in 20 mM propionate, wt *Mtb* was severely impaired for growth, whereas *MtbΔwhiB3* growth appeared unaffected (Fig. 5B and inset). This striking phenotype was reversed by complementation of *MtbΔwhiB3* with an intact copy of *whiB3* (Fig. 5). These observations suggest that increased resistance of *MtbΔwhiB3* to toxic levels of propionate could be due to channeling of propionate into PDIM via the methyl-malonyl CoA (MMCoA) pathway, and TAG.

### *Mtb* WhiB3 is essential for maintaining intrabacterial redox homeostasis during infection

Having established that the synthesis of virulence lipids is directly mediated by WhiB3 in a redox-dependant manner, we now sought to examine the defined role of WhiB3 in maintaining the *Mtb* intracellular redox environment. Since the pyridine nucleotides (NAD^+^ and NADH) and their phosphorylated forms (NADP^+^ and NADPH) are central to catabolic and anabolic metabolism, respectively, we utilized a recently developed [^14^C] nicotinamide incorporation assay [22] and measured the redox poise of NADH or NADPH from *Mtb* cells cultured *in vitro*, or derived from macrophages (see Protocol S1).

**Figure 6:**
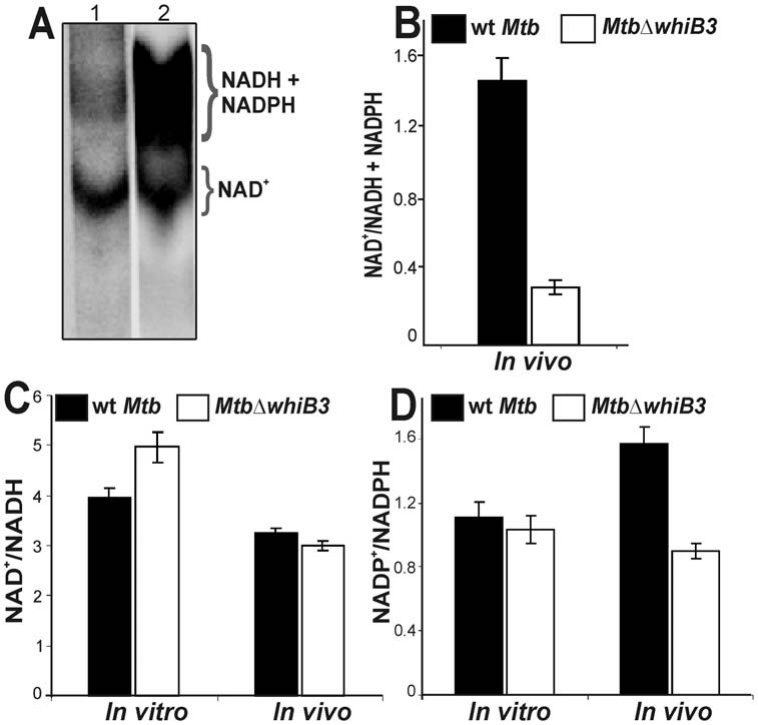
*Mtb* WhiB3 maintains the intrabacterial NAD^+^/NADH and NADP^+^/NADPH poise. (A) TLC analysis of oxidized and reduced pyridine nucleotides labeled with [^14^C] nicotinamide. Wt *Mtb* (lane 1) and *MtbΔwhiB3* (lane 2) cells isolated from infected macrophages (∼10^8^ cells) were labeled with [^14^C] nicotinamide for 24 h in 7H9 basal medium containing acetate as a carbon source and analyzed by TLC by loading equal cpm in each lane. Note the strong labeling of reduced pyridine nucleotides from *MtbΔwhiB3* isolated from macrophages. (B) Densitometric analysis of the relative abundance of nucleotides in (A). Experiments were performed four times and similar observations were recorded. Cells grown *in vitro* or isolated from the infected macrophages were analyzed by enzymatic assays using (C) alcohol dehydrogenase for NAD^+^/NADH estimation and (D) glucose-6-phosphate dehydrogenase for NADP^+^/NADPH analysis. Data shown is the average of two independent experiments.

Since the *Mtb* NAD salvage pathway is not efficient *in vitro* [22], we observed minor incorporation of nicotinamide into NAD^+^ of wt *Mtb* and *MtbΔwhiB3* cultured *in vitro* (results not shown). However, a significant increase in the incorporation of exogenously added nicotinamide into NAD^+^ in wt *Mtb* cells from infected macrophages was observed (Fig. 6A lane 1, B). Furthermore, a dramatic increase in the labeling of the band that corresponds to NADH and/or NADPH from *MtbΔwhiB3* cells within macrophages was observed (Fig. 6A lane 2, 6B). Consistent with previous reports [23], using different solvents, separation of NADH and NADPH by TLC was ineffective. Regardless, these results demonstrate that *MtbΔwhiB3* accumulates significant quantities of NADH and/or NADPH within macrophages, and therefore experiences considerable reductive stress.

In order to determine the exact contribution of NADH and NADPH in the above radiolabeling experiments, we exploited an enzymatic cycling assay to distinguish between NADH and NADPH. We cultured *Mtb* derived from macrophages in 7H9 basal medium containing acetate as a sole carbon source [19]. We observed a small, but reproducible increase in NAD^+^/NADH from *MtbΔwhiB3* cells grown *in vitro* as compared to wt *Mtb* (Fig. 6C), suggesting efficient recycling of NADH because of increased acetate metabolism. This is consistent with our previous findings [19] that *MtbΔwhiB3* grow better on acetate containing media than wt *Mtb*. Surprisingly, the increased NAD^+^/NADH ratio in *MtbΔwhiB3* was restored to wt *Mtb* levels during growth in macrophages (Fig. 6C). Since NADPH is an essential reductant used in lipid anabolism [24], we sought to examine the influence of defective lipid anabolism in *MtbΔwhiB3* on the redox poise of NADP^+^/NADPH. During growth *in vitro*, the NADP^+^/NADPH ratio remained the same (Fig. 6D). However, we observed a ∼2-fold reduction in the NADP^+^/NADPH ratio from *MtbΔwhiB3* cells growing within macrophages as compared to wt *Mtb* (Fig. 6D), demonstrating that *MtbΔwhiB3* accumulates substantial levels of NADPH. The radiolabel and enzymatic cycling assays are in reasonable agreement. However, although widely used, the acidic/alkaline extraction method used in the enzymatic assay is associated with a loss or oxidation of reduced pyridine nucleotides. Therefore, the radiolabeling method is a much more accurate indicator of the intrabacterial redox poise. In sum, these results suggest that *MtbΔwhiB3* experiences reductive stress during infection, and that the WhiB3-dependant redox regulation of virulence lipids is essential for maintaining intrabacterial redox homeostasis during macrophage infection.

### *MtbΔwhiB3* modulates the innate immune response

**Figure 7:**
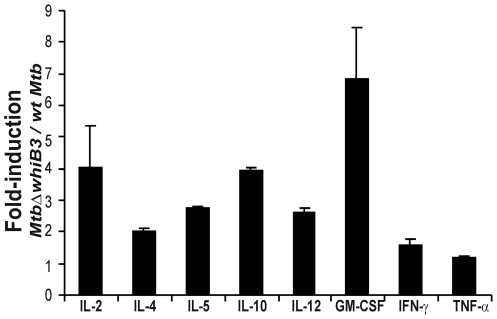
*MtbΔwhiB3* alters macrophage inflammatory cytokine production. Raw 264.7 macrophages were infected with well dispersed cells of wt *Mtb* and *MtbΔwhiB3* at a MOI of 10∶1 for 24 h and the concentration of cytokine in the supernatant representing the Th1 and Th2 immune responses were quantified using the Bio-Plex multiplex Human Cytokine Th1/Th2 Assay kit (BioRad Laboratories).

*Mtb* cell wall lipids are direct modulators of the host immune response. Our current and previous [13] results strongly suggest that the WhiB3-mediated redox regulation of virulence lipids may influence macrophage response. Here we sought to examine and compare the release of pro and anti-inflammatory cytokines from macrophages infected with wt *Mtb* and *MtbΔwhiB3*. We infected macrophages with well-dispersed *MtbΔwhiB3* cells to completely rule out the influence of aggregation on infection. We analyzed the levels of IL-2, IL-4, IL-5, IL-10, IL-12(p70), GM-CSF, IFN-γ and TNF-α in the culture supernatant of macrophages 24 h post infection. Both strains grew at a similar rate within macrophages (data not shown) and induce the release of various cytokines in the culture supernatant 24 h post infection. However, *MtbΔwhiB3* elicited significantly higher levels of pro- and anti-inflammatory cytokines as compared to wt *Mtb* (Fig. 7). Thus, in spite of an identical number of intracellular bacteria, macrophages infected with *MtbΔwhiB3* produced higher amounts of cytokines, and this provides further proof that the immune suppressing capacity of *Mtb* is impaired in *MtbΔwhiB3*. The data suggest that the WhiB3-mediated regulation of complex lipids, and perhaps other *Mtb* factors, [25] modulates the kinetics and balance between pro- and anti-inflammatory cytokines to favor long term persistence of the bacilli, which may explain the *in vivo* phenotype exhibited by *MtbΔwhiB3* [13].

### Mechanism of WhiB3 mediated regulation of lipid biosynthesis

Having fully confirmed the role of WhiB3 in regulating cell wall lipid biosynthesis *in vitro* and in macrophages, we now sought to examine the underlying molecular and biochemical mechanisms. For more than 15 years WhiB homologues in actinomycetes have been speculated to be putative redox- responsive DNA-binding transcription factors [26]. However, formal proof demonstrating their DNA binding activity in a redox-dependent manner is lacking. In the sections below, we first describe how the expression of polyketide genes was altered in the *MtbΔwhiB3*. We then demonstrate the influence of the redox state of the 4Fe-4S cluster on DNA binding of reconstituted WhiB3 (otherwise referred to as holo-WhiB3). Furthermore, we then examine how changes in the Cys thiol oxidation state affect DNA binding of apo-WhiB3 (without 4Fe-4S cluster). Lastly, we analyzed apo- and holo-WhiB3 DNA binding properties.

### *Mtb* WhiB3 directly controls the expression of polyketide biosynthetic genes

**Figure 8:**
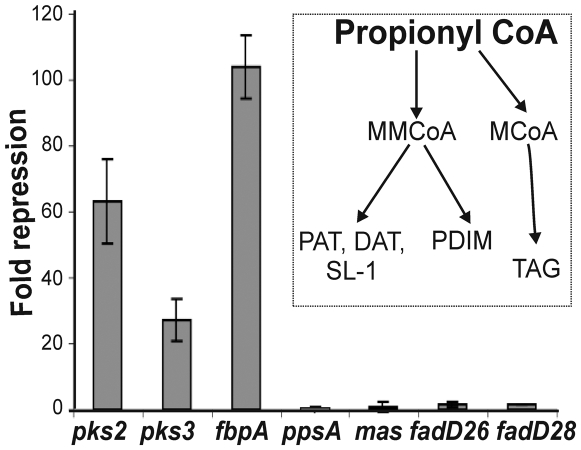
*Mtb* WhiB3 regulates the expression of polyketide biosynthetic genes. Total RNA was isolated from logarithmically grown cells of wt *Mtb* and *MtbΔwhiB3* and the expression of PAT, DAT, SL-1 and PDIM biosynthetic genes was analyzed by real time PCR. Inset; the pathway describing the incorporation of a common precursor propionyl-CoA into PAT, DAT, SL-1, PDIM and TAG. RT-PCR data suggests that the repression of PAT, DAT and SL-1 biosynthetic genes in *MtbΔwhiB3* results in the accumulation of propionyl CoA, which is then diverted to PDIM and TAG production.

Here we examine whether WhiB3 regulates the production of complex lipids by controlling the expression of genes encoding modular polyketide synthases. Since the identity of genes required for SL-1, PAT, DAT, TDM and PDIM are well established, we used quantitative PCR (Q-PCR) and analyzed the expression of *pks2* (necessary for SL-1 production), *pks3* (necessary for PAT/DAT production), *fbpA* (necessary for TDM production), *mas*, *ppsA*, *fadD26* or *fadD28* (necessary for PDIM production) in wt *Mtb* and *MtbΔwhiB3* cells. Consistent with our lipid data (Fig. 2A–E), we found that *pks2*, *pks3* and *fbpA* expression were down-regulated 62.5, 32.8 and 111-fold, respectively, in *MtbΔwhiB3* (Fig. 8). In sum, our data suggest that WhiB3 is a positive transcriptional regulator of genes responsible for the production of PAT, DAT, SL-1 and TMM/TDM. However, we did not detect any significant changes in the transcript levels of genes involved in the biosynthesis of PDIM. This suggest a post-transcriptional mode of regulation for PDIM, perhaps by escalating PDIM production to compensate for increasing propionyl CoA and/or methyl malonyl CoA (MMCoA) levels caused by inefficient utilization via PAT/DAT and SL-1 (Fig. 8 inset). These results are consistent with a recent study linking the regulation of SL-1 and PDIM biosynthesis with a common precursor, MMCoA [20].

### *Mtb* WhiB3 is a DNA binding protein

**Figure 9:**
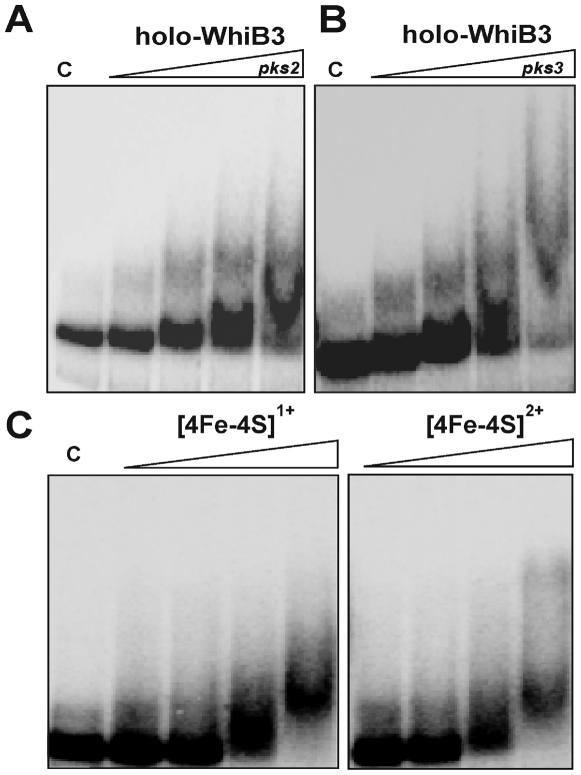
*Mtb* WhiB3 is a Fe-S cluster containing DNA binding protein. The [4Fe-4S]^2+^ form of WhiB3 was utilized for EMSA. EMSA reactions were performed under anaerobic conditions using 0.2 nM ^32^P labeled (A) *pks2*, or (B) *pks3* promoter DNA. The WhiB3 [4Fe-4S]^2+^ concentrations used for EMSA were 0, 100, 200, 400 and 800 nM. (C) The redox status of the 4Fe-4S cluster does not influence WhiB3 DNA binding. Identical concentrations (800 nM) of WhiB3 [4Fe-4S]^2+^ and dithionite-reduced WhiB3 [4Fe-4S]^1+^ were analyzed for their ability to bind *pks3* promoter DNA.

Having established that WhiB3 regulates the production of major complex polyketides and lipids of *Mtb*, we next sought to determine if WhiB3 could directly bind to the promoter regions of *Mtb pks2* and *pks3*. Interestingly, WhiB3 overexpressed and purified from *E. coli* was always associated with contaminating genomic DNA. Subsequently, we took thorough measures to ensure complete removal of DNA prior to performing DNA binding studies (See Protocol S1). The WhiB3 [4Fe-4S]^2+^ cluster was effectively reconstituted *in vitro* using NifS and confirmed by UV-Vis spectroscopy (Fig. S5) as described previously [19]. Holo-WhiB3 was then analyzed for DNA binding activity under anaerobic conditions. The data demonstrate that WhiB3 binds to the promoter regions (∼300 bp upstream from ATG) of both *pks2* and *pks3* (Fig. 9A and 9B) in a concentration dependent manner. When incubated with 400 nM and 800 nM holo-WhiB3, a diffused smear was observed, suggesting weak DNA binding activity. The weak DNA binding acitivty of holo-WhiB3 was confirmed by performing EMSA analyses in the presence of a NaCl gradient (see below). In sum, this data demonstrate that *Mtb* holo-WhiB3 possesses DNA binding activity.

### The redox state of the WhiB3 4Fe-4S cluster does not influence DNA binding

Previously, we have shown that the WhiB3 [4Fe-4S]^2+^ cluster can easily be reduced to [4Fe-4S]^1+^ by the one electron reducing agent dithionite [19]. Since it is well known that the redox status of Fe-S clusters of regulatory proteins can affect DNA binding (*e.g.*, SufR; [27]) or transcriptional activation (*e.g.*, SoxR; [28]), we sought to examine the effect of the redox status of the WhiB3 Fe-S cluster on DNA binding. Reduction of the WhiB3 [4Fe-4S]^2+^ cluster to a [4Fe-4S]^1+^ cluster by DTH was confirmed by UV-vis spectroscopy as described [19]. Reduced ([4Fe-4S]^1+^) and oxidized ([4Fe-4S]^2+^) holo-WhiB3 fractions were purified via size exclusion chromatography and analyzed for *pks3* promoter DNA binding under anaerobic conditions. As shown in Fig. 9C, reduced and oxidized holo-WhiB3 binds to the *pks3* promoter at similar concentrations. Also, we did not observed any noticeable difference in the mobility of WhiB3 [4Fe-4S]^2+^:DNA and WhiB3 [4Fe-4S]^1+^:DNA complexes, strongly suggesting that the redox state of the WhiB3 4Fe-4S cluster does not modulate DNA binding.

### The redox status of the WhiB3 Cys residues modulates DNA binding

It is known that despite containing redox active 4Fe-4S cluster, aconitase binds specific mRNAs in the apo-form [29]. Furthermore, the RNA binding activity of aconitase is regulated by the redox state of its Cys residues. Since O_2_ destroys WhiB3 Fe-S cluster to generate apo-protein containing exposed Cys residues [19], we sought to examine apo-WhiB3 and the influence of the redox state of its Cys residues on DNA binding.

**Figure 10:**
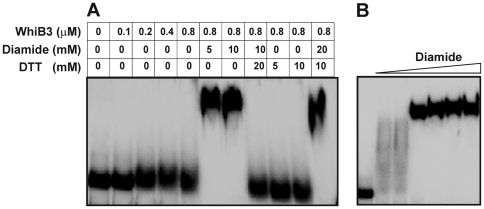
A thiol-disulfide redox switch reversibly regulates WhiB3 DNA binding. (A) The inhibitory effect of DTT on apo-WhiB3 binding to *pks3* promoter DNA is reversed by diamide. (B) Dose-dependent effect of diamide on DNA binding. EMSA was performed using diamide (50, 100, 200, 400, 800 and 1000 µM), *pks3* promoter DNA, and apo-WhiB3 (800 nM).

Apo-WhiB3 was generated as described [30]. Complete loss of Fe-S cluster from WhiB3 was confirmed by UV-Vis spectroscopy and mass spectrometry (data not shown). We thoroughly deoxygenated apo-WhiB3 and pre-incubated with diamide (a thiol-specific oxidant) or DTT (a thiol-specific reductant) and examined the protein∶DNA complexes by electromobility shift assays (EMSA) under fully anaerobic conditions. Fig. 10A shows that apo-WhiB3 treated with diamide (WhiB3-SS) rapidly induces the formation of a WhiB3-SS:DNA complex of retarded mobility (Fig. 10A). However, in the case of DTT-treated WhiB3 (apo-reduced; WhiB3-SH), we observed complete inhibition of DNA-binding (Fig. 10A). Furthermore, DNA binding was rapidly restored when WhiB3-SH was exposed to diamide. Similarly, DNA binding was lost when WhiB3-SS was treated with DTT (Fig. 10A). Lastly, we showed that under these experimental conditions, >100 µM diamide is sufficient to induce apo-WhiB3 DNA binding (Fig. 10B). The above results suggest that oxidation of WhiB3 Cys thiols stimulates apo-WhiB3 DNA binding, but that reduction of the WhiB3 thiols abolish DNA binding. Consistent with the above findings, our *in vitro* thiol trapping experiment confirmed that diamide induces oxidation of the four WhiB3 Cys residues to generate two intramolecular disulphide bonds (Fig. S6). In sum, our results demonstrate that the WhiB3 Cys center functions as a thiol-based nano-switch that modulates DNA binding.

### WhiB3 DNA binding and sequence specificity

**Figure 11:**
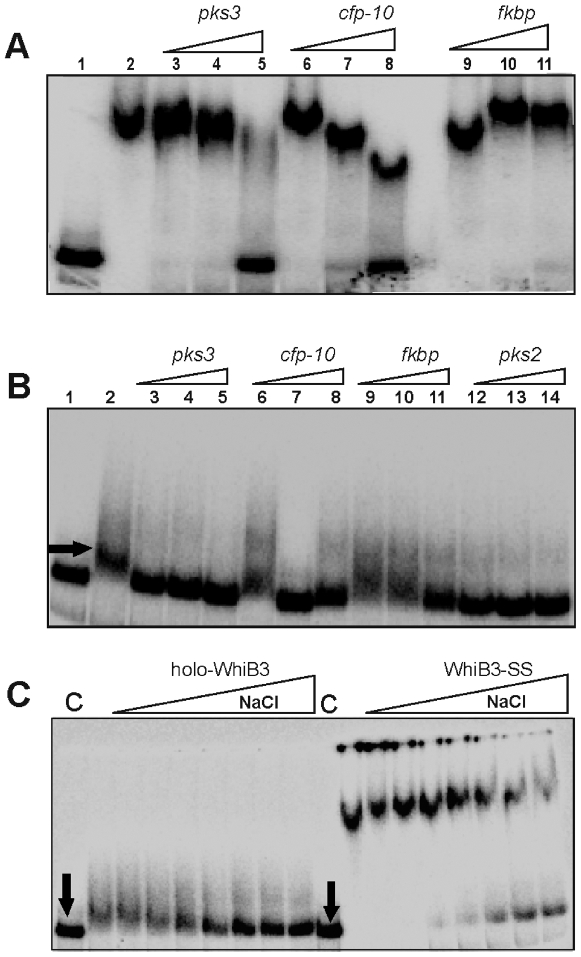
WhiB3 DNA binding and sequence specificity. (A) Sequence specificity of WhiB3-SS DNA binding. 0.2 nM ^32^P labeled *pks3* promoter DNA with 800 nM of WhiB3-SS were used in the EMSA analysis. Lane 1; free probe, lane 2; WhiB3-SS:DNA complex. WhiB3 DNA binding was competed using 200 (lane; 3, 6 and 9), 400 (lane; 4, 7 and 10) and 800 (lane; 5, 8 and 11) fold molar excess of unlabeled competitor DNA [*pks3* (specific), *cfp-10* (non-specific), mammalian *FKBP* ORF (non-specific)]. (B) Lane 1; free probe, lane 2; holo-WhiB3:DNA complex. Competition assay for sequence specificity of holo-WhiB3 (800 nM) using *pks3* promoter (specific, lane; 3, 4 and 5), *cfp-10* ORF (non- specific, lane; 6, 7 and 8), mammalian *fkbp* ORF (non- specific, lane; 9, 10 and 11), and *pks2* promoter (specific, lane, 12, 13 and 14) as competitor DNA. These results demonstrate that WhiB3 has low sequence discrimination. Our unpublished data have shown that WhiB3 binds strongly to linear and supercoiled pUC19 DNA, as well as *Hind*III digested λ-DNA. This, together with the *cfp-10*, *ftsQ*, and *FKBP* control DNA binding experiments provides strong evidence for the non-specific DNA binding activity of WhiB3. Arrow indicates the minor retardation of *pks3* promoter due to weak binding of holo-WhiB3. (C) Oxidized apo-WhiB3 (WhiB3-SS) binds DNA stronger than holo-WhiB3. NaCl was used to examine and compare the contribution of the [4Fe-4S]^2+^ cluster or disulphide bonds on the strength of DNA (*pks3* promoter) binding. EMSA was performed under anaerobic conditions using 800 nM holo-WhiB3 or WhiB3-SS in the presence of 0, 100, 200, 300, 400, 600, 800, 1000 mM of NaCl. C; DNA binding without NaCl. Arrows; free probe. In each experiment, a promoter fragment containing 330 bp sequences upstream of *pks3* ATG was used as radiolabeled probe.

In order to study the specificity of WhiB3 binding to the radiolabeled *pks3* promoter, several DNA fragments were utilized in the competition assays, (i) specific *Mtb* DNA comprised of the *pks3* and *pks2* promoter region, (ii) non-specific *Mtb* open reading frame (ORF) DNA (Rv3874, *cfp-10*) and (iii) non-specific eukaryotic ORF DNA encoding FK506 binding protein (FKBP). First, we performed competition assays to examine the sequence specificity of WhiB3-SS DNA binding. Fig. 11A demonstrated that competition with an 800-fold molar excess of specific unlabeled *pks3* DNA resulted in complete loss of WhiB3-SS DNA-binding whereas the same concentration of non-specific eukaryotic DNA, *fkbp*, caused no loss of DNA binding. Interestingly, we observed that an 800-fold molar excess of mycobacterial ORF DNA, *cfp-10*, resulted in significant, but not complete loss of WhiB3 DNA binding (Fig. 11A). Furthermore, our results showed that competition with *cfp-10* generates a sharp band of progressively changing mobility, suggesting discrete structural complexes of different mobilities. Similar findings were obtained using the specific *Mtb pks2* promoter region and non-specific *Mtb* ORF DNA (Rv2151c, *ftsQ*) (results not shown).

Next, an identical set of experiments were performed as described in Fig. 11A, except that holo-WhiB3 was used. As anticipated, Fig. 11B shows that holo-WhiB3 retarded DNA marginally as compared to WhiB3-SS. Furthermore, a 200-fold molar excess of specific unlabeled *pks3* or *pks2* DNA resulted in complete loss of DNA binding, whereas, a 400-fold (*cfp-10*) to 800-fold (*fkbp*) excess of non-specific competitor DNA caused a complete loss of holo-WhiB3:DNA binding. Since, holo and apo-WhiB3 generate distinct complexes, our results suggest that differences in oligomerization influence their mobilities. .Lastly, to analyze strength of apo- and holo-WhiB3 DNA, we performed EMSA in the presence of a NaCl gradient. As expected, 400–600 mM of NaCl completely abolished holo-WhiB3 DNA binding whereas 1 M of NaCl was insufficient to prevent apo-WhiB3 DNA binding (Fig. 11C). These results confirmed that WhiB3-SS binds DNA significantly stronger than holo-WhiB3.

In sum, the data generated by several independent experiments demonstrate that WhiB3 is a redox-responsive DNA binding protein, and that WhiB3-SS and holo-WhiB3 bind specific and non-specific DNA with a low degree of discrimination

## Discussion

Members of the WhiB-like protein family play an important role in actinomycete biology and pathogenesis. However, a detailed biochemical and genetic mechanism of WhiB function has not yet been established. In this study we provide unique insight into the mechanism of action of *Mtb* WhiB3 and show that WhiB3 regulates the production of the inflammatory polyketides PAT, DAT, SL-1 and PDIM, and lipid inclusion bodies (TAG) via a novel, redox-dependent switching mechanism. We developed a methodology to characterize lipid profiles of *Mtb* residing in macrophages and demonstrated that intrabacterial redox homeostasis is maintained by WhiB3 in part via channeling reducing equivalents into *Mtb* lipid synthesis, which modulate inflammatory cytokine production. These findings, as well as the discovery of a link between the intracellular WhiB3 virulence pathway and extracellular DosR/S/T dormancy signaling pathway, significantly impact our understanding of the role of WhiB3 in *Mtb* pathogenesis and persistence. Our findings introduce a new mechanism, which suggests that WhiB3 modulates *Mtb* lipid anabolism in response to oxido-reductive stress associated with infection to maintain redox balance and persistence.

Previously, we proposed that WhiB3 senses fluctuations in the intracellular redox state of *Mtb* in response to O_2_ depletion and fatty acid metabolism, and maintains redox balance by regulating polyketide anabolism [19]. Surprisingly, the role of oxidative and/or reductive stress in regulating *Mtb* lipid anabolism has not yet been described and represents an understudied area of TB research. The absence of PAT, DAT and SL-1, and accumulation of PDIM and TAG in *MtbΔwhiB3* strongly suggests a role for WhiB3 in regulating lipid anabolism in response to the redox imbalance associated with normal cellular metabolism. Importantly, the fact that the expression of genes responsible for PDIM production is not differentially regulated in *MtbΔwhiB3* suggests a post-transcriptional mode of regulation for PDIM biosynthesis, which is in agreement with a recent study suggesting the regulation of SL-1 and PDIM biosynthesis by a common precursor, MMCoA [20]. According to this model, inhibition of the PDIM pathway leads to accumulation of MMCoA, which is then diverted towards the synthesis of SL-1 [20]. However, disruption of the SL-1 pathway did not result in the accumulation of PDIM [20], suggesting that other factors are involved. Our WhiB3 data provides new insight into this important central metabolic branch point, suggesting that joint inhibition of all three methyl branched lipids (PAT, DAT and SL-1), as opposed to SL-1 alone, is required to accumulate suitable levels of MMCoA to enhance PDIM levels (Fig. 8 inset). Furthermore, since *MtbΔwhiB3* also accumulates TAG (which utilizes malonyl CoA rather than MMCoA), this suggests a central role for propionyl CoA rather than MMCoA in the synthesis of PAT, DAT, SL-1, PDIM and TAG (Fig. 8, inset). The *Mtb* response regulator PhoP, which responds to a yet-to-be identified environmental signal, has been shown to positively regulate the production of PAT, DAT and SL-1 [31]. However, the expression of *phoP* is not altered in *MtbΔwhiB3* (unpublished observation) and *Mtb*Δ*phoP* did not accumulate PDIM or TAG, suggesting that the WhiB3-dependent control of cellular redox homeostasis is an additional factor that is required to regulate the flux of propionyl CoA to methyl-branched polyketides and TAG synthesis. Thus, we discovered a new redox switching mechanism by which *Mtb* differentially assimilates fatty acid (propionate) into PDIM, TAG, SL-1 and PAT under defined oxidizing and/or reducing conditions *in vitro*. The physiological relevance of these redox-related metabolic events was supported by directly examining the lipid profiles of *Mtb* in resting macrophages (Fig. 4).

Using a combination of sensitive metabolic labeling techniques and high resolution mass spectrometry, we demonstrated that WhiB3 is a major redox regulator of pathogenic lipid anabolism *in vitro* and within macrophages. During macrophage infection, wt *Mtb* predominantly assimilate propionate into methyl-branched polyketides (PAT and SL-1) and surprisingly, also into the TAG biosynthetic pathway. Minor induction of PDIM biosynthesis was also observed. In contrast, *MtbΔwhiB3* mainly accumulates PDIM and TAG inside the macrophages. These results are in agreement with a recent intraphagosomal microarray data demonstrating synchronized induction of WhiB3 with the genes responsible for the production of PAT, DAT, SL-1 and TAG [17], supporting the role of WhiB3 as a physiological regulator of *Mtb* lipids *in vivo*.

Since *Mtb* subsists on fatty acids as a primary carbon source *in vivo* [32], it is believed that persistent *Mtb* not only requires efficient metabolism of fatty acid oxidation intermediates *(e.g.*, propionate) as a energy source, but also their detoxification [21]. The regulatory mechanism controlling *Mtb* growth and survival in response to accumulation of toxic levels of propionate is not known. The increased resistance of *MtbΔwhiB3* towards toxic concentrations of propionate, and the induction of PDIM and TAG production in *MtbΔwhiB3* in macrophages, strongly suggests that the WhiB3-mediated regulation of polyketide/lipid anabolism represents a mechanism for the detoxification of accumulated propionate metabolites *in vivo*. These findings provide new insight into the mechanisms of virulence and long-term persistence *in vivo*. For example, two recent reports suggested that *in vivo* persistence of *Mtb* hinges on methyl-branched polyketide anabolism, which alleviates the potential toxic effect of propionate accumulation during growth on odd chain fatty acids as carbon source [20],[33]. Our findings suggest that *Mtb* has evolved an efficient genetic and metabolic circuit operated by WhiB3 to effectively coordinate propionate flux into methyl-branched fatty acids and TAG, which is necessary for growth on fatty acids.

The link between lipid anabolism and intrabacterial redox balance was further substantiated by directly measuring the accumulation of significant quantities of NADPH and/or NADH in *MtbΔwhiB3* isolated from macrophages (Fig. 5A–D). NADPH is an essential cofactor for lipid anabolism and varying levels of lipid synthesis will influence the NADP^+^/NADPH poise. Consistent with this, our results suggest that the upregulation of methyl-branched lipids and TAG in wt *Mtb* results in efficient consumption of NADPH, and is therefore associated with an increased NADP^+^/NADPH ratio within macrophages. However, since the PAT and SL-1 pathways are absent in *MtbΔwhiB3*, attempts to restore physiological levels of NADPH via increased production of PDIM and TAG appears to be partially successful and resulted in amplified levels of NADPH and NADH. Since TAG expression and production [9] is also induced upon exposure to NO, CO (which inhibits respiration) and hypoxia via the DosR/S/T dormancy system, our results establish a novel association between TB dormancy signals, NADPH accumulation (reductive stress) and TAG production. Nonetheless, we do not exclude other mechanisms that may also modulate reductive stress. For example, NADPH accumulation could be due to alterations in mycothiol disulfide (MSSM) reduction by NADPH-dependent mycothiol-reductase, or changes in the expression/activity of pyridine nucleotide transhydrogenase (SthA), which is responsible for generating NADPH. This is currently under investigation.

Since it has been suggested that host fatty acid catabolism provides precursors (*e.g.*, propionate etc) for *Mtb* lipid anabolism [20], our data also provides new insight into this mechanism by suggesting that essential reductants (NADH) are generated by β-oxidation of host lipids, which are required for *Mtb* lipid anabolism. Consistent with this view, Boshoff et al., [22], have demonstrated dramatic accumulation of *Mtb* NADH and/or NADPH during infection *in vivo*, thereby providing unambiguous evidence of a role for reductive stress in *Mtb* pathogenesis.

Our findings point to a general, albeit important role for *Mtb* lipid anabolism as a mechanism for relieving propionate toxicity as proposed earlier [33] as well as dissipating excess reducing equivalents.

**Figure 12:**
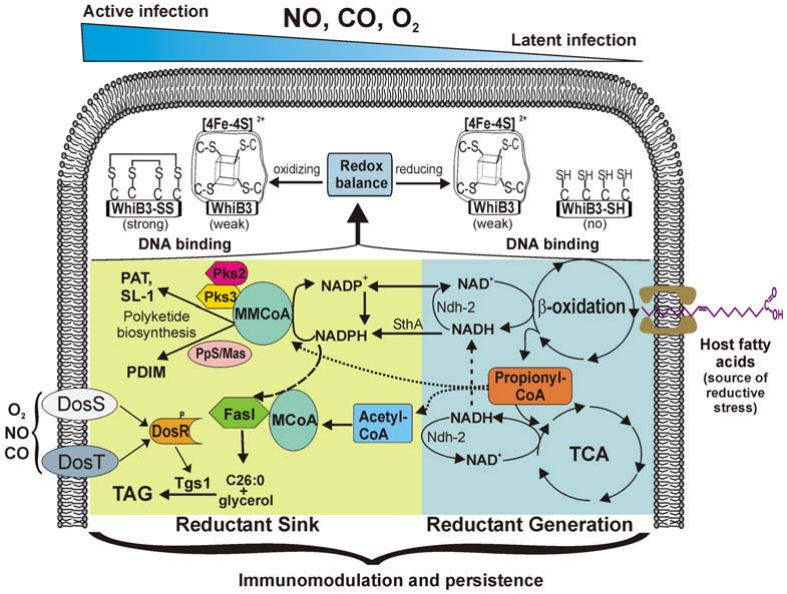
The reductive stress dissipation model for *Mtb* persistence. Since gradients of NO, CO, O_2_ and host fatty acids generated within microenvironments of the lungs are most likely encountered by *Mtb* during infection [48], we propose that an intracellular *Mtb* redox imbalance is caused by the catabolism of highly reducing host lipids, which influences endogenous *Mtb* polyketide anabolism. In this model the DNA binding activity of WhiB3 is activated by post-translational modifications of the WhiB3 Cys thiols in response to redox stress to generate a distinct cellular response, which modulates the production of inflammatory polyketides and storage lipids (TAG) to maintain intracellular redox homeostasis, and to modulate virulence. A crucial component of the model is the strong reducing power of host fatty acids, and inhibition of respiration by NO, CO and hypoxia, which results in the accumulation of reducing equivalents in *Mtb* to generate reductive stress. In order to dispose of excess reductants, the bacilli anabolize PAT, SL-1, PDIM and TAG, which function as an electron sink. Notably, reductive stress is further enhanced by NO, CO, or hypoxia. Since TAG is also induced upon exposure to NO, CO and hypoxia via the DosR/S/T dormancy system, our results suggest cross-talk between the WhiB3 and Dos dormancy pathways resulting in TAG anabolism to dissipate reducing equivalents. The lipid defects exhibited by *MtbΔwhiB3* during infection suggest that WhiB3 functions as a nano-switch to systematically regulate the production of virulence lipids to maintain redox homeostasis and persistence.

This mechanism has obvious *in vivo* relevance since it is widely accepted that *Mtb* switches from carbohydrates to host fatty acids in the phagosome [32]. An overlooked, albeit well-known physicochemical feature is that long chain host fatty acids (*e.g.*, palmitate [C_16_H_32_O_2_]) have highly reduced oxidation states (carbon oxidation state = −28, 106 ATP) compared to glucose (carbon oxidation state = 0, 38 ATP) and upon β-oxidation cause a substantial cellular redox imbalance favoring the production of NADH, which generates reductive stress. Ironically, the production of ROI is increased through the auto-oxidation of NADH [34], which amplifies oxidative stress. This apparent counterintuitive concept has significant implications for understanding oxidative stress, which can be prevented by efficient disposal of excess reductants. Bacterial disposal mechanisms of excess reductants include using nitrate as terminal electron acceptor under anaerobic conditions, the reductive fixation of CO_2_ (via the reductive TCA cycle), and the use of NADH to reduce metabolic intermediates (fermentation). However, none have yet been demonstrated in *Mtb*. Reductive stress has a clear bearing on bacterial pathogenesis *e.g.*, under nitrosative stress, *S. aureus* starts fermenting to dispose of excess NADH [35], whereas *Pseudomonas spp*. maintain cellular redox balance by secreting redox active polyketides (phenazines) to oxidize accumulating NADH [36]. Since most human pathogens have to subsist on an *in vivo* nutrient (*e.g.*, carbon) source, and are exposed to NO and/or CO, our findings may serve as a model foundation for understanding how pathogens respond to environments *in vivo* that generates intrabacterial reductive stress. Although the detailed mechanism remains to be established (currently in progress), we anticipate that the newly discovered link between oxido-reductive stress, lipid anabolism and *Mtb* persistence described in this study invites new and unexplored avenues of future research. In Fig. 12 we propose a “reductive stress dissipation model” for WhiB3 redox regulation.

The Th1 and Th2 cytokine data (Fig. 6) demonstrate that *MtbΔwhiB3* enhances both the pro- and anti-inflammatory immune response. Since careful dosing of a Th1 and Th2 response is essential for controlling *Mtb* infection without causing detrimental immunopathology [37], the disturbed kinetics and balance between Th1 and Th2 cytokines caused by the loss of *whiB3* could in part explain the unique *in vivo* phenotype exhibited by *MtbΔwhiB3* [13]. This is not unprecedented, since live cells or lipids derived from *Mtb* CDC1551 induced higher levels of Th1 and Th2 cytokines and exhibits an altered immuno-pathology [38], a phenotype also exhibited by *MtbΔwhiB3* [13].

The fact that WhiB3 modulates polyketide production under oxidative and reductive stress and is required for the expression of *pks2*, *pks3* and *fbpA* suggest a role for WhiB3 as a redox-dependent transcription factor. To explore the molecular mechanism underlying WhiB3 function, we extensively characterized its DNA binding properties under various redox conditions and resolved a long-standing issue whether the WhiB-like proteins can bind DNA. We provide conclusive evidence that at least one WhiB member binds DNA, and that WhiB3 is the first redox-dependent DNA binding protein identified in *Mtb*.

In the case of metal-based sensors, either the presence (*e.g* FNR) or redox state (*e.g* SufR) of an Fe-S cluster regulates DNA binding [27],[29]. However, thiol-based sensors exploit a thiol-disulfide redox switch (*e.g.* OxyR, CrtJ) to modulate DNA binding [29]. Interestingly, despite possessing a Fe-S cluster, the RNA binding activity of aconitase is regulated by the redox state of its thiols [39]. On the other hand, to the best of our knowledge, methodical studies examining the effect of the redox state of apo-FNR or apo-SoxR Cys thiols on DNA binding are fragmentary. Our findings demonstrating that the redox state of the 4Fe-4S cluster has virtually no effect on the ability of holo-WhiB3 to bind DNA, whereas post-translational modification of the Cys thiols significantly stimulates WhiB3 DNA binding, are unique. This is reminiscent of OxyR where similar thiol-based post-translational modifications influence DNA binding and transcription activation properties to generate graded transcriptional responses [40]. Although DNA binding does not reflect transcriptional activation, it is tempting to speculate that the WhiB3 thiol modifications also differentially affect transcription. A characteristic that may shed light on the biological function of WhiB3 is the poor discrimination between specific and non-specific DNA binding. A clear DNA binding (*e.g.* HTH) domain is absent in WhiB3. However, WhiB7 [16] and WhiB3 contain characteristic AT-hook motifs RPRGRPRKDAVA and TMGRTRGIRRTA, respectively, at their C-termini. These motifs are found in several non-specific DNA binding proteins with weak specificity such as the high-mobility group (HMG) non-histone nuclear proteins. Similar to histones in chromatin, architectural bacterial proteins such as Lrp, and H-NS bind DNA specifically and non-specifically [41]. However, the non-specific and specific DNA binding of the various redox forms of WhiB3 requires further investigation and is the focus of an independent study.

In sum, our data establishes a paradigm for WhiB-like proteins in maintaining redox homeostasis. In particular, our data implicate WhiB3 in sensing reductive stress generated by host lipid catabolism. Importantly, *Mtb* WhiB3 functions as an intracellular redox sensor by controlling the flux of lipid precursors and reducing equivalents through the biosynthesis of virulence polyketides and storage lipids necessary for achieving redox balance and to modulate host innate immunity.

## Materials and Methods

### Bacterial strains and growth conditions

*M. tuberculosis* H37Rv, *MtbΔwhiB3*, and *MtbΔwhiB3 tetRO:whiB3* [19] were grown at 37°C in 7H9 (broth) or 7H11 (agar) media with 1xADS enrichment (albumin-dextrose-NaCl), 0.05% glycerol and 0.1% Tween 80 (broth). *E. coli* cultures were grown in LB medium. Antibiotics were added as described earlier [19]. For propionate toxicity assays, bacteria were grown in 7H9 broth containing 0.5% albumin, 0.085% NaCl, 0.02% tyloxypol and 10 mM or 20 mM sodium propionate as the carbon source.

### Electron microscopy

*Mtb* strains were grown to stationary phase (10 days growth) and cells were analyzed by SEM and TEM as previously described [42].

### Biochemical analysis of cell wall lipids

Metabolic radiolabeling of complex cell wall lipids of *Mtb* were performed as previously described [31]. In short, *Mtb* were cultured to OD_600 nm_ = 1.5 in 20 ml of 7H9 medium followed by addition of 20 µCi of [1, 2-^14^C] sodium acetate (for labeling mycolic acids) or [1-^14^C] sodium propionate (for labeling methyl branched lipids) and incubating for 24 h. When necessary, 5 mM diamide or DTT were added to the culture along with the corresponding radiolabeled lipid precursor. Cultures were centrifuged, and washed once in distilled water. Cell wall surface lipids were extracted first with CHCl_3_/CH_3_OH (1∶2, v/v) for 24 h, and then with CHCl_3_/CH_3_OH (2∶1, v/v), for 48 h. The organic phases were pooled, washed twice with distilled water and dried. Mycolic acid methyl esters (MAME) were prepared from [1, 2-^14^C] acetic acid labeled *Mtb* cells by extraction with 20% tetrabutylammonium hydroxide (Sigma-Aldrich) at 100°C in an oil bath. This was followed by methylation using methyl iodide, extraction with dichloromethane, and finally drying under nitrogen. The crude lipid extracts were analyzed by TLC on precoated silica plates (F_254_; Sigma-Aldrich) in different solvent systems. Radiolabeled lipids were visualized by autoradiography of the TLC plates using phosphoimaging. The utilization of solvent systems to identify various lipids based on the retention factor (*Rf*) by TLC was performed as described [5],[43],[44].

### Metabolic labeling and extraction of bacterial lipids from mouse macrophages

Raw264.7 cell lines (5×10^8^) were infected in quadruplicate using DMEM medium containing 10% FCS at a multiplicity of infection (MOI) of 10∶1 with various strains and incubated for 4 h for internalization. Infected macrophages were washed thrice with warm DMEM medium followed by the addition of fresh medium and 50 µCi of sodium propionate and incubation was continued for 2 days. Two days post-infection macrophages were washed and harvested in PBS. Macrophage-derived lipids were removed by suspending infected macrophages in 10 ml methanol, followed by vortexing (thrice for 10 s each), and centrifugation (4,000 rpm for 10 min). This step was repeated twice. This was followed by extraction of mycobacterial lipids with 10 ml of chloroform∶methanol (2∶1) for 48 h.

### FT-ICR MS analysis of mycobacterial lipids

Lipids were isolated from *Mtb* inside macrophages as described above and analyzed by FT-ICR MS as described [20]. Lipids were suspended in choloroform∶methanol (2∶1), in 0.1% formic acid. A monolithic microchip-based electrospray interface, the TriVersa NanoMate (Advion, Ithaca, NY) served as the ESI source as previously reported [45],[46]. The NanoMate was set to load 5 µl of sample which was electrosprayed in negative ion mode by applying −1.8 kV spray voltage and 0.2 psi nitrogen head pressure to the sample tip to obtain a constant spray for 15–20 min. The capillary temperature, spray voltage, and tube lens voltage were set to 200 C, −36 V, and −100 V, respectively. Mass spectra were acquired by use of a hybrid two dimensional linear quadrupole ion trap Fourier transform ion cyclotron resonance mass spectrometer (LTQ FT MS, Thermo Fisher Scientific, San Jose, CA). The mass spectrometer was operated in the high mass range to obtain negative ion FT-ICR mass spectra (1550<*m/z*<3500) at a mass resolving power of 100,000 at *m/z* 400 and a automatic gain control (AGC) target value of 2×10^6^, maximum fill 200 ms. Each displayed spectrum represents a sum of 30–50 scans.

### Estimation of NADH and NADPH

For the *in vivo* estimation of pyridine nucleotides, we infected Raw264.7 macrophages with wt *Mtb* and *MtbΔwhiB3* for 2 days. Preparation of *Mtb* cells and pyridine nucleotide analysis were performed as described [47] except that 5 mM acetate was used in the growth incubation step. A similar approach was recently utilized to study salvage pathway involved in NAD^+^ and NADH synthesis in *Mtb* growing *in vivo* [22]. As an *in vitro* control, *Mtb* strains were cultured directly in 7H9 medium containing 5 mM acetate and pyridine nucleotides were estimated. Assaying for NADPH was performed in a similar manner, except that glucose-6-phosphate dehydrogenase and glucose-6-phosphate was utilized as enzyme and substrate, respectively.

### [^14^C] Nicotinamide incorporation assay

Raw264.7 macrophages were infected with various strains for 2 days as described in the previous section. Bacteria derived from macrophages were grown in 7H9 basal medium containing 5 mM acetate as the sole carbon source for 24 h and 20 µCi of [^14^C] nicotinamide was added for labeling of NAD^+^ and NADH (American Radiolabeled chemicals, Inc). Labeled nucleotides were extracted and analyzed by TLC analysis as described [22].

### Th1/Th2 Bio-Plex cytokine assay

Raw264.7 cell lines were infected in triplicate as described in the previous section. Supernatants from the infected macrophages was harvested 24 h post-infection and subjected to cytokine analysis using the Bio-Plex multiplex Human Cytokine Th1/Th2 Assay kit (Bio-Rad) and the Cytokine Reagent kit (Bio-Rad) in accordance with the manufacturer's protocols.

### Q-PCR

*Mtb* cells were harvested and RNA was isolated as described [48]. First-strand synthesis was performed by using 500 ng total RNA with iScript Select cDNA Synthesis Kit (Bio-Rad) using random oligonucleotides. PCR was performed using gene specific primers. Expression of genes was analyzed with real-time PCR using iQ SYBR Green Supermix (Bio-Rad) and a BioRad iCycler 5 with an iQ Multicolor Real-Time PCR Detection System (Bio-Rad). Data analysis was performed with the iQ Multicolor Real-Time PCR Detection System Optical Software System (Bio-Rad), version iQ5. PCR efficiencies were normalized to obtain accurate expression levels. For comparisons between wt *Mtb* and *MtbΔwhiB3*, the induction ratio for each gene was normalized to *Mtb* 16s rRNA expression.

### Overexpression and purification of *Mtb* WhiB3

*Mtb* WhiB3 was overexpressed in *E. coli* and purified as described in Protocol S1.

### Iron-sulfur cluster assembly

WhiB3 Fe-S cluster assembly was performed under anoxic conditions and monitored by UV-visible spectroscopy as described previously [19] (see also Protocol S1).

### Preparation of redox-modified forms of apo-WhiB3

Apo-WhiB3 was generated as previously described [30]. Reduced apo-WhiB3 (WhiB3-SH) was generated by addition of 5 to 20 mM of DTT for 30 min, followed by size exclusion chromatography inside an anaerobic glove box. Oxidized WhiB3 (WhiB3-SS) was generated by removal of DTT from apo-WhiB3 by size exclusion chromatography followed by treating samples with 5 to 20 mM diamide for 30 min.

### EMSA analysis

For EMSA assays, promoter fragments (∼300 bp upstream of translational start codon) of *pks2*, and *pks3* were PCR amplified from the *Mtb* H37Rv genome and the 5′-end labeled with [γ-^32^P] ATP (GE Healthcare) using T4 polynucleotide kinase (MBI Fermentas) according to the manufacturer's instructions. Binding of WhiB3 to *pks3* or *pks2* promoters were performed inside a PlasLabs anaerobic glovebox. Reactions were performed in buffer containing 89 mM Tris, 89 mM boric acid and 1 mM EDTA, pH 8.4 in the presence of 50 µg of salmon sperm DNA. WhiB3, DNA and buffers were completely degassed using argon gas. Aliquots were incubated for 30 min at room temperature. The reactions were separated inside an anaerobic glovebox using 4–20% gradient TBE PAGE gels (Bio-Rad). Gels were exposed to autoradiographic film and visualized via phosphorimaging (GE).

## Supporting Information

Protocol S1 Supporting materials and methods.
(0.03 MB PDF)

Figure S1 *Mtb* WhiB3 regulates methyl-branched polyketide lipid production in early logarithmic phase. Wt *Mtb* and *MtbΔwhiB3* were cultured in 7H9 medium to OD_600 nm_ = 0.6 and total lipids were labeled for 24 h using [^14^C] propionate. Silica TLC plates were loaded with 100,000 cpm of total lipids. PAT (A) and PDIM (B) polyketide lipids were resolved using petroleum ether∶acetone (92∶8) and petroleum ether∶ethyl acetate (98∶2, two developments), respectively. Lane 1: wt *Mtb* and lane 2: *MtbΔwhiB3*.
(2.57 MB TIF)

Figure S2 *MtbΔwhiB3* accumulates TAG in response to reductive stress. Total lipids were labeled using 1–2 [^14^C] acetate under oxidizing (5 mM diamide) and reducing (5 mM DTT) conditions. In each case, equal count (100,000 cpm) was separated by TLC using n-hexane/diethyl ether (90∶10) as solvent. Note the increase of TAG in DTT treated *MtbΔwhiB3*.
(2.63 MB TIF)

Figure S3 *Mtb* WhiB3 mediated synthesis of SL-1 in macrophages. Metabolically labeled total lipids from *in vivo* (macrophages) and *in vitro* (7H9 medium) grown wt *Mtb* and *MtbΔwhiB3* cells were extracted and analyzed for SL-1 by spotting total lipids (50,000 cpm) on a silica TLC plate with chloroform∶methanol (90∶10) as solvent. Note the accumulation of highly polar lipids at the origin of the *MtbΔwhiB3* lanes.
(3.81 MB TIF)

Figure S4 FT-ICR mass spectra of total crude lipids derived from *MtbΔwhiB3* growing in macrophages demonstrate the absence of SL-1. (A) Total crude lipid extracts were prepared from *Mtb* growing inside macrophages and analyzed in the negative ion mode by FT-ICR MS. We observed the presence of Ac_2_PI species at *m/z* 835.5261 and 851.5566, which corresponds to their reported theoretical masses. The dimannose specie esterified to three acyl chains (Ac_3_PIM_2_) corresponds to mass *m/z* 1413.8888 was also detected in all the strains tested. Note that the multiple lipoforms of SL-1 were absent in *MtbΔwhiB3*. (B) The SL-1 region of FT-ICR mass spectrum showed a complete absence of this class of lipids (∼*m/z* 2300 to 2600) in *MtbΔwhiB3* (C) FT-ICR mass spectra of the PDIM region. Note that although PDIM lipid species are present in all three strains, FT-ICR (as opposed to radiolabeling) does not allow the quantification of these species. Compl; *MtbtetRO:whiB3*.
(8.63 MB TIF)

Figure S5 Spectroscopic characterization of WhiB3 Fe-S reconstitution. Reconstitution was carried out inside an anaerobic glovebox as described previously [1]. At the indicated time points, samples were scanned using UV-visible spectroscopy. Note the time-dependent increase in the characteristic 4Fe-4S absorption peak at 413 nm.
(8.09 MB TIF)

Figure S6 Intramolecular disulphide bond formation of apo-WhiB3. We used *in vitro* thiol-trapping experiments to specifically examine the role of the WhiB3 Cys residues in disulphide bond formation. Iodoacetamide (IAM) forms a covalent adduct with the free sulfhydryl group of Cys to increase the molecular mass by 57 Da/Cys thiol. We independently exposed apo-WhiB3 to diamide and DTT, followed by alkylation using IAM of the respective samples. Aliquots were then analyzed by MALDI-TOF. In the case of (B) DTT exposed apo-WhiB3 (WhiB3-SH) treated with IAM, we observed a major peak at 14636.76, whereas (A) diamide exposed WhiB3 generated peaks at ∼14407. The mass difference of 229.54 Da between reduced and oxidized WhiB3 suggests that all four Cys thiols were alkylated after reduction with DTT and that all four Cys residues are engaged in intramolecular disulphide bond formation upon diamide oxidation.
(4.18 MB TIF)
