# Supplementary material for: Mycobacterium tuberculosis WhiB3 Maintains Redox Homeostasis by Regulating Virulence Lipid Anabolism to Modulate Macrophage Response
Source: PLoS Pathog. 2009 Aug 14;5(8):e1000545. doi: 10.1371/journal.ppat.1000545 (PMC2718811; doi:10.1371/journal.ppat.1000545)
Supplement: Protocol S1 — Supporting materials and methods. (0.03 MB PDF) [file ppat.1000545.s001.pdf]

SI Materials and Methods

Q-PCR

Mtb cells were harvested and RNA was isolated as described (2). First-strand synthesis was performed by using 500 ng total RNA with iScript Select cDNA Synthesis Kit (Bio-Rad) using random oligonucleotides. PCR was performed using gene specific primers. Expression of genes was analyzed with real-time PCR using iQ SYBR Green Supermix (Bio-Rad) and a BioRad iCycler 5 with an iQ Multicolor Real-Time PCR Detection System (Bio-Rad). Data analysis was performed with the iQ Multicolor Real-Time PCR Detection System Optical Software System (Bio-Rad), version iQ5. PCR efficiencies were normalized to obtain accurate expression levels. For comparisons between wt *Mtb* and *MtbΔwhiB3*, the induction ratio for each gene was normalized to *Mtb* 16s rRNA expression.

Overexpression and purification of *Mtb* WhiB3

Mtb WhiB3 ORF (Rv3416) was PCR amplified using complementary oligonucleotides harboring *Bgl*II and *Xho*I restriction enzymes sites at their 5` and 3` ends. PCR fragments was treated with *Bgl*II and *Xho*I and ligated into a similarly modified *E. coli* expression vector, pETDuet (Novagen) to generate pETDuet-W3. The pETDuet-W3 was transformed into *E. coli* Rossetta (Novagen) cells. Transformants were grown at 37°C in LB broth containing ampicillin to an A_{600}nm of 0.6 and expression was induced with 0.8 mM of IPTG for 5 h at 37°C. *Mtb* WhiB3 expressed under these growth conditions were found to be in inclusion bodies. WhiB3 was purified from inclusion bodies by washing twice with 50 ml of sodium phosphate buffer, pH 7.5, 300 mM NaCl and 0.1 x Triton followed by three washes with 50 ml of sodium phosphate buffer, pH 7.5 and 300 mM NaCl. Inclusion bodies were treated with 5M urea, 3M NaCl in 50 mM sodium phosphate buffer, pH 7.5 for ~2h at 37°C with slow stirring to completely remove genomic DNA associated with WhiB3. Inclusion bodies were pelleted by centrifugation at 18,000 rpm for 30 min, and further solubilized in buffer A (8 M urea, 50 mM sodium phosphate, pH 7.5, 300 mM NaCl and 5 mM DTT) for 16 h at 37°C. Denatured WhiB3 was renatured by step-wise dialysis against buffer B (4 M urea, 50 mM sodium phosphate, pH 7.5, 300 mM NaCl and 5 mM DTT) for 6 h, followed by buffer C (2 M urea, 50 mM sodium

phosphate, pH 7.5, 300 mM NaCl and 5 mM DTT) for 6 h, and finally in buffer D (50 mM sodium phosphate, pH 7.5, 300 mM NaCl, 5 mM DTT and 10 % glycerol). Refolded WhiB3 was concentrated using iCON concentrators (PIERCE) and stored at –20°C. WhiB3 was also purified from soluble fraction as a SUMO-fusion as described previously (1). WhiB3 purity (~90%) was judged by Coomassie Blue staining of an SDS-PAGE gel and by MALDI-TOF mass spectrometry. Our UV-visible spectroscopic results showed no difference in the redox sensing properties between purified WhiB3 fused with S- or SUMO-tag. Subsequently, because of its small size, the WhiB3 S-tagged protein was used in all studies.

Preparation of apo-WhiB3

The 4Fe-4S cluster in holo-WhiB3 was removed by incubating protein with EDTA and potassium ferricyanide in a molar ratio of protein:EDTA:ferricyanide in 1:50:20 at room temperature. Loss of 4Fe-4S cluster was monitored over time by UV-vis spectroscopy. Finally, apo-WhiB3 was subjected to gel-exclusion chromatography to remove low molecular weight materials. Purified apo –WhiB3 was used for *in vitro*-thiol trapping and DNA binding experiments.

***In vitro*- thiol trapping and MALDI-TOF analysis**

Apo-WhiB3 (10 μM) was first treated with 2 mM of DTT for 1 h at room temperature followed by labeling of free thiols by treating with 20 mM IAM for 1 h in dark at room temperature. The diamide oxidized apo-WhiB3 were similarly treated with IAM and taken as a control. All the samples were precipitated with 10 % trichloroacetic acid (TCA) for 30 min on ice, washed with acetone, air dried. Samples were analyzed in the positive mode on a Voyager Elite mass spectrometer with delayed extraction technology (PerSeptive Biosystems, Framingham MA). The acceleration voltage was set at 25kV and 10-50 laser shots were summed. Sinapinic acid (Aldrich, D13, 460-0) dissolved in acetonitrile: 0.1% TFA (1:1) was the matrix used. The mass spectrometer was calibrated with apomyoglobin. Samples were diluted 1:10 with matrix, and 1 ul was pipetted on to a smooth plate.

SI note 1.

Currently, there is no known method for effectively measuring intrabacterial redox homeostasis (via NAD/NADH and/or NADP/NADPH) in infected macrophages. Nonetheless, Boshoff *et al* (3) developed an "*in vivo*" method that utilizes *Mtb* cells derived from macrophages, or mouse lungs, followed by a 24 h incubation step in which [C^{14}] nicotinamide is incorporated into NAD/NADH and/or NADP/NADPH. It is well-known that the *Mtb* NAD salvage pathway is not efficient during *in vitro* growth (3). However, the method developed by Boshoff *et al.*, clearly demonstrated that the NAD salvage pathway is switched on *in vivo* and remains functional in *Mtb* cells derived from macrophages even when cultured for 24 h *in vitro*. As a result, [C^{14}] nicotinamide incorporation into NAD, followed by endogenous reduction to generate NADH, is an effective indicator of the intrabacterial redox poise.

References

1. Singh, A., Guidry, L., Narasimhulu, K. V., Mai, D., Trombley, J., Redding, K. E., Giles, G. I., Lancaster, J. R., Jr. & Steyn, A. J. (2007) *Mycobacterium tuberculosis* WhiB3 responds to O2 and nitric oxide via its [4Fe-4S] cluster and is essential for nutrient starvation survival. *Proc Natl Acad Sci U S A* **104,** 11562-7.
2. Kumar, A., Toledo, J. C., Patel, R. P., Lancaster, J. R., Jr. & Steyn, A. J. (2007) *Mycobacterium tuberculosis* DosS is a redox sensor and DosT is a hypoxia sensor. *Proc Natl Acad Sci U S A* **104,** 11568-73.
3. Boshoff, H. I., Xu, X., Tahlan, K., Dowd, C. S., Pethe, K., Camacho, L. R., Park, T. H., Yun, C. S., Schnappinger, D., Ehrt, S., Williams, K. J. & Barry, C. E., 3rd (2008) Biosynthesis and recycling of nicotinamide cofactors in *Mycobacterium tuberculosis*. An essential role for NAD in nonreplicating bacilli. *J Biol Chem* **283,** 19329-41.
