# Supplementary figures and images for: Mycobacterium tuberculosis WhiB3 Maintains Redox Homeostasis by Regulating Virulence Lipid Anabolism to Modulate Macrophage Response
Source: PLoS Pathog. 2009 Aug 14;5(8):e1000545. doi: 10.1371/journal.ppat.1000545 (PMC2718811; doi:10.1371/journal.ppat.1000545)

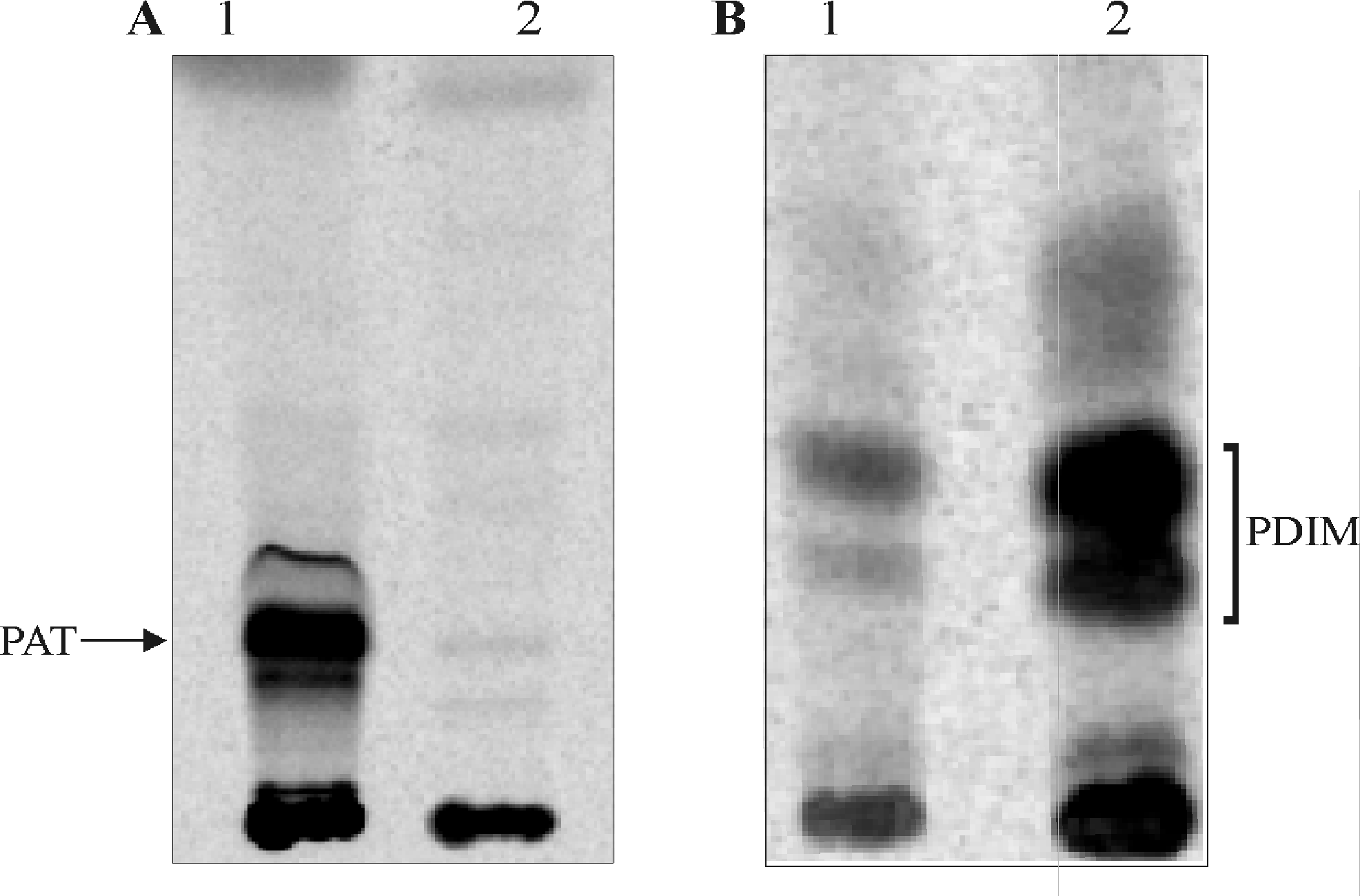

Supplement: Figure S1 — Mtb WhiB3 regulates methyl-branched polyketide lipid production in early logarithmic phase. Wt Mtb and MtbΔwhiB3 were cultured in 7H9 medium to OD600 nm = 0.6 and total lipids were labeled for 24 h using [14C] propionate. Silica TLC plates were loaded with 100,000 cpm of total lipids. PAT (A) and PDIM (B) polyketide lipids were resolved using petroleum ether∶acetone (92∶8) and petroleum ether∶ethyl acetate (98∶2, two developments), respectively. Lane 1: wt Mtb and lane 2: MtbΔwhiB3. (2.57 MB TIF) [file ppat.1000545.s002.tif]

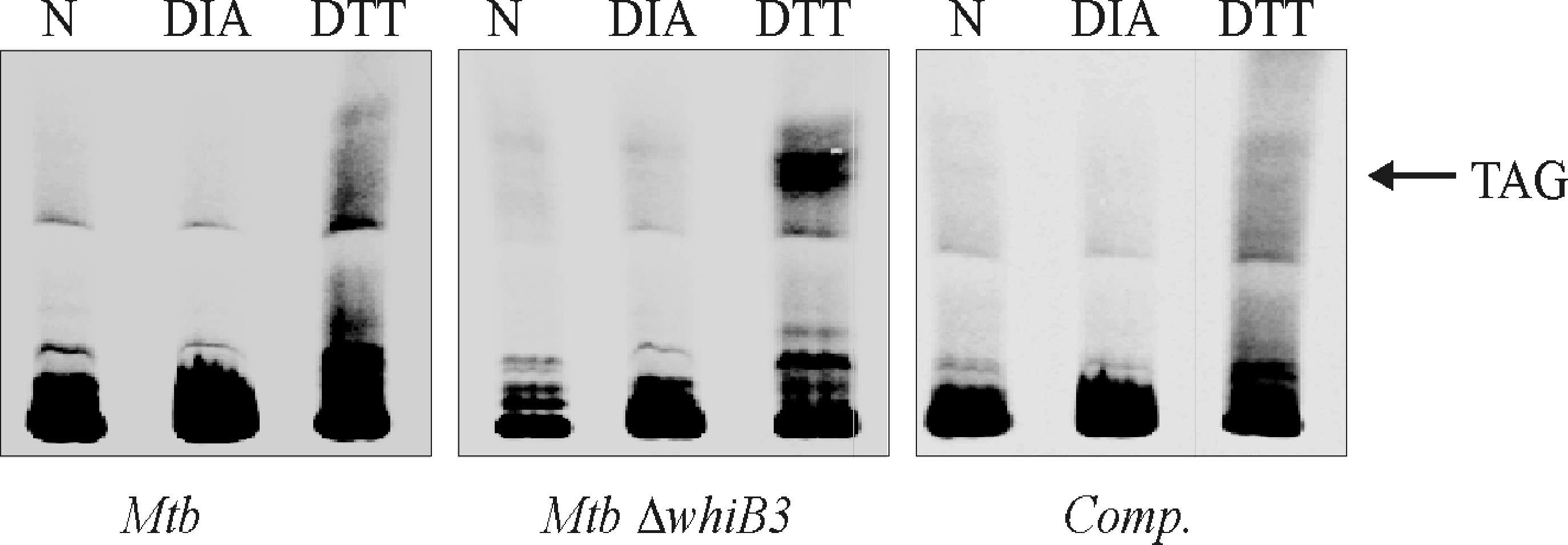

Supplement: Figure S2 — MtbΔwhiB3 accumulates TAG in response to reductive stress. Total lipids were labeled using 1–2 [14C] acetate under oxidizing (5 mM diamide) and reducing (5 mM DTT) conditions. In each case, equal count (100,000 cpm) was separated by TLC using n-hexane/diethyl ether (90∶10) as solvent. Note the increase of TAG in DTT treated MtbΔwhiB3. (2.63 MB TIF) [file ppat.1000545.s003.tif]

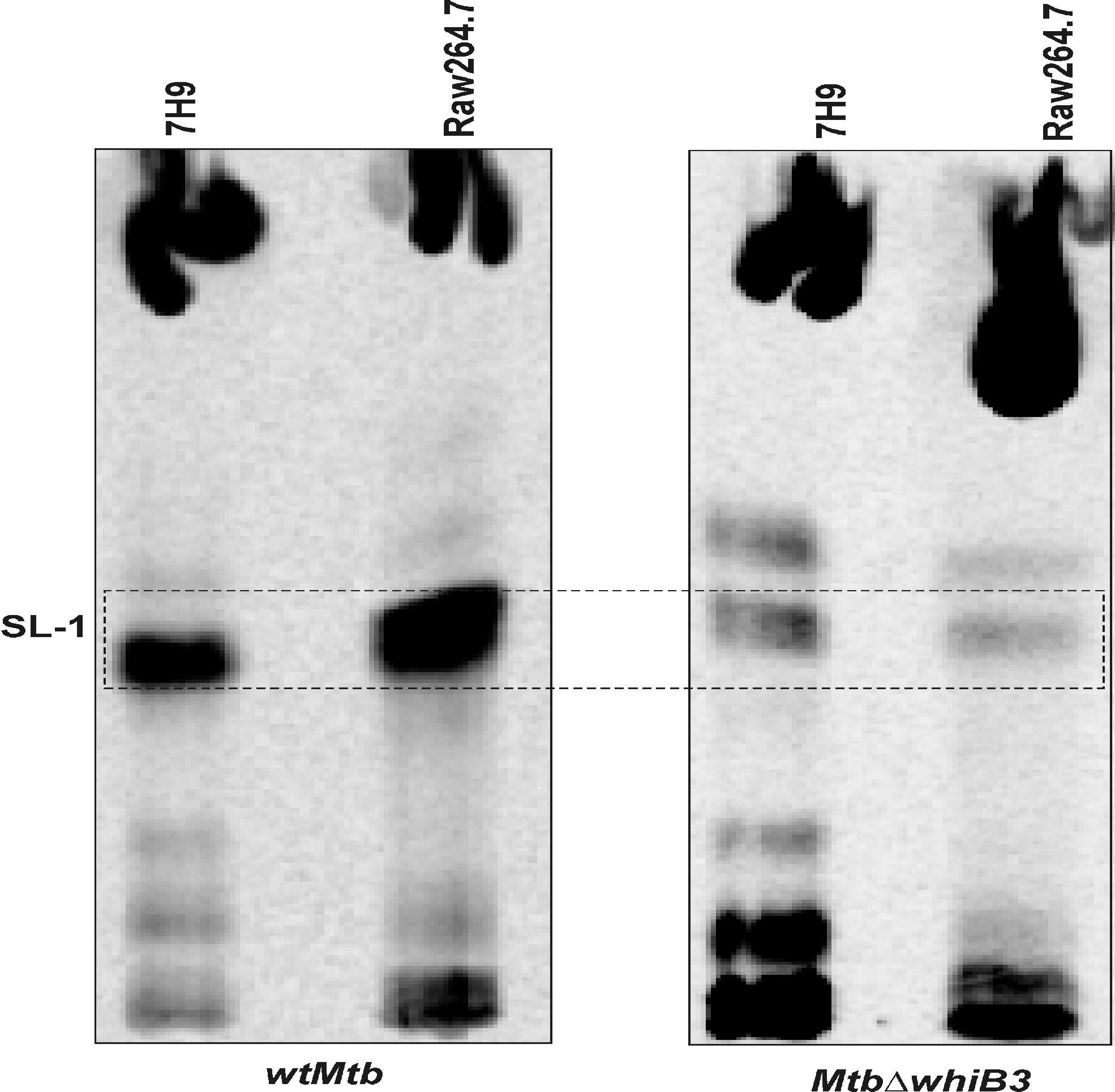

Supplement: Figure S3 — Mtb WhiB3 mediated synthesis of SL-1 in macrophages. Metabolically labeled total lipids from in vivo (macrophages) and in vitro (7H9 medium) grown wt Mtb and MtbΔwhiB3 cells were extracted and analyzed for SL-1 by spotting total lipids (50,000 cpm) on a silica TLC plate with chloroform∶methanol (90∶10) as solvent. Note the accumulation of highly polar lipids at the origin of the MtbΔwhiB3 lanes. (3.81 MB TIF) [file ppat.1000545.s004.tif]

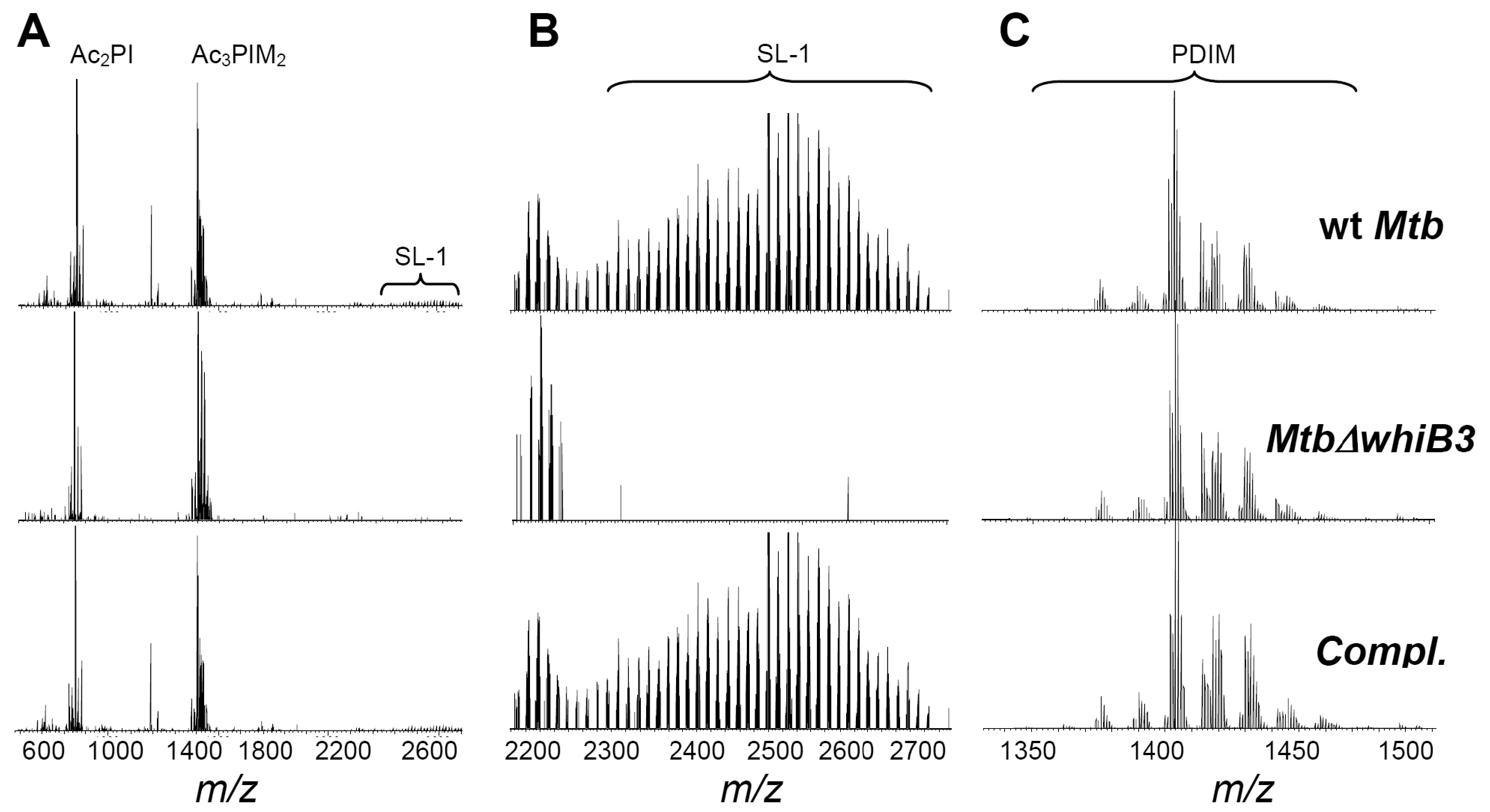

Supplement: Figure S4 — FT-ICR mass spectra of total crude lipids derived from MtbΔwhiB3 growing in macrophages demonstrate the absence of SL-1. (A) Total crude lipid extracts were prepared from Mtb growing inside macrophages and analyzed in the negative ion mode by FT-ICR MS. We observed the presence of Ac2PI species at m/z 835.5261 and 851.5566, which corresponds to their reported theoretical masses. The dimannose specie esterified to three acyl chains (Ac3PIM2) corresponds to mass m/z 1413.8888 was also detected in all the strains tested. Note that the multiple lipoforms of SL-1 were absent in MtbΔwhiB3. (B) The SL-1 region of FT-ICR mass spectrum showed a complete absence of this class of lipids (∼m/z 2300 to 2600) in MtbΔwhiB3 (C) FT-ICR mass spectra of the PDIM region. Note that although PDIM lipid species are present in all three strains, FT-ICR (as opposed to radiolabeling) does not allow the quantification of these species. Compl; MtbtetRO:whiB3. (8.63 MB TIF) [file ppat.1000545.s005.tif]

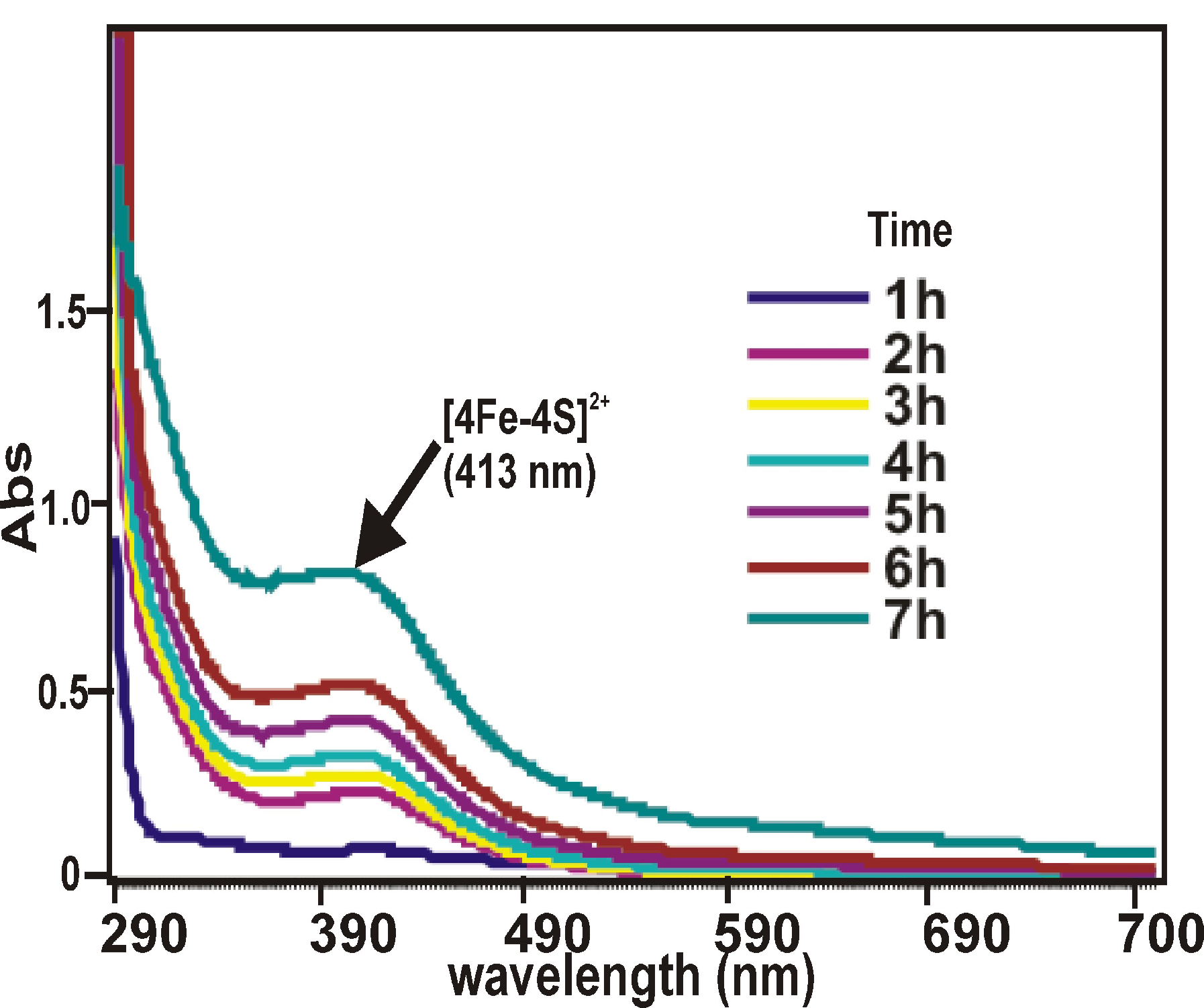

Supplement: Figure S5 — Spectroscopic characterization of WhiB3 Fe-S reconstitution. Reconstitution was carried out inside an anaerobic glovebox as described previously [1]. At the indicated time points, samples were scanned using UV-visible spectroscopy. Note the time-dependent increase in the characteristic 4Fe-4S absorption peak at 413 nm. (8.09 MB TIF) [file ppat.1000545.s006.tif]

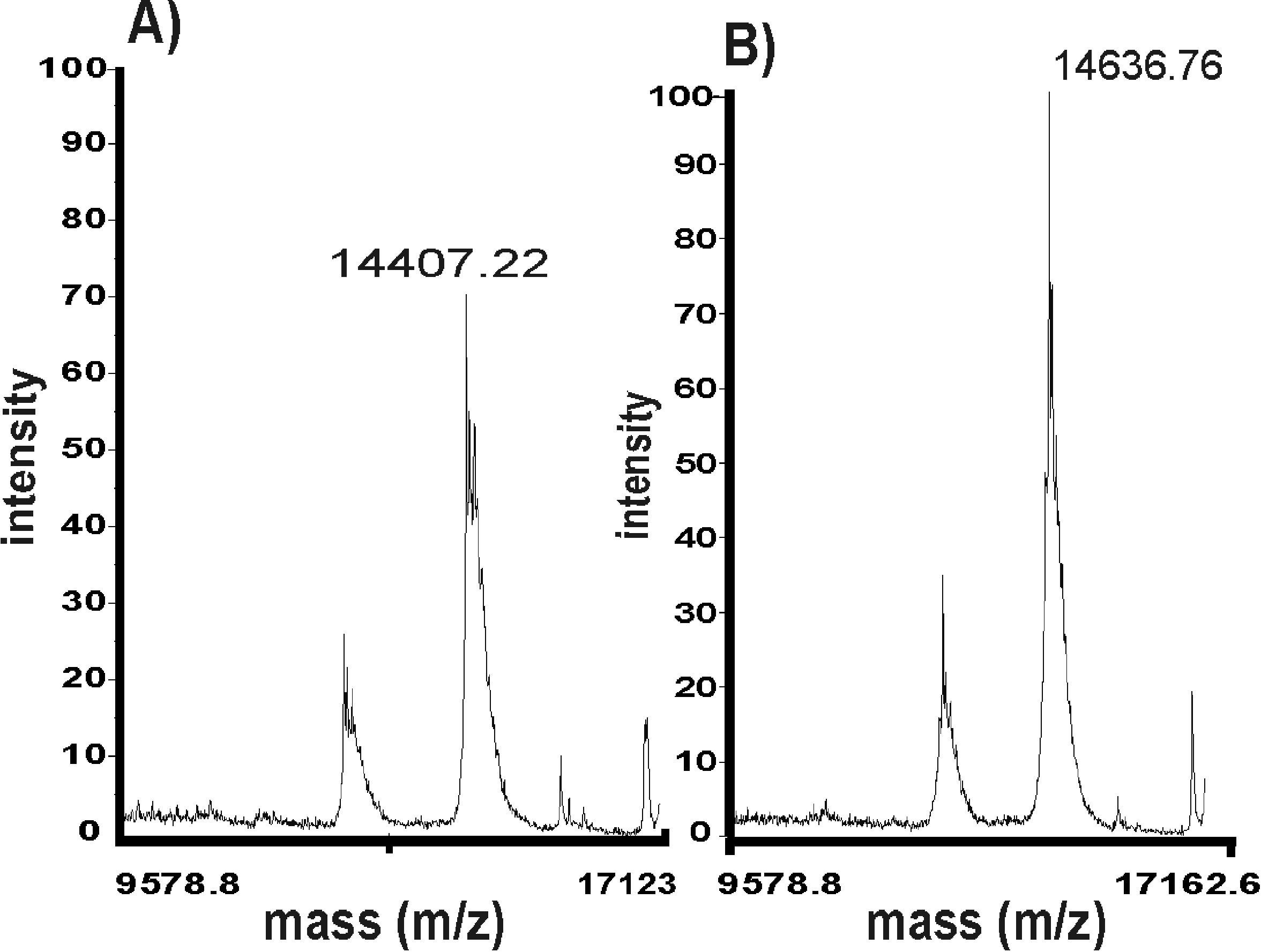

Supplement: Figure S6 — Intramolecular disulphide bond formation of apo-WhiB3. We used in vitro thiol-trapping experiments to specifically examine the role of the WhiB3 Cys residues in disulphide bond formation. Iodoacetamide (IAM) forms a covalent adduct with the free sulfhydryl group of Cys to increase the molecular mass by 57 Da/Cys thiol. We independently exposed apo-WhiB3 to diamide and DTT, followed by alkylation using IAM of the respective samples. Aliquots were then analyzed by MALDI-TOF. In the case of (B) DTT exposed apo-WhiB3 (WhiB3-SH) treated with IAM, we observed a major peak at 14636.76, whereas (A) diamide exposed WhiB3 generated peaks at ∼14407. The mass difference of 229.54 Da between reduced and oxidized WhiB3 suggests that all four Cys thiols were alkylated after reduction with DTT and that all four Cys residues are engaged in intramolecular disulphide bond formation upon diamide oxidation. (4.18 MB TIF) [file ppat.1000545.s007.tif]
